# ﻿Discovering fungal communities in roots of *Zoysiajaponica* and characterising novel species and their antifungal activities

**DOI:** 10.3897/imafungus.16.138479

**Published:** 2025-02-19

**Authors:** Haifeng Liu, Hyeongju Choi, Narayan Chandra Paul, Hiran A. Ariyawansa, Hyunkyu Sang

**Affiliations:** 1 Department of Integrative Food, Bioscience and Biotechnology, Chonnam National University, Gwangju, Republic of Korea; 2 Kumho Life Science Laboratory, Chonnam National University, Gwangju, Republic of Korea; 3 Department of Plant Pathology and Microbiology, National Taiwan University, Taipei, Taiwan

**Keywords:** Bioactivity, endophytic fungi, ITS amplicons, phylogeny, turf-grass

## Abstract

Turf-grasses are economically important horticultural crops, which have been utilised by humans to improve the environment for more than a thousand years. Turf-grasses are widely distributed in landscapes, slopes and sport fields, such as golf courses. Endophytic fungi are a resource of unexplored fungal diversity with potential bioactive compounds. In this study, culture-independent ITS amplicon sequencing and culture-dependent isolation methods were used to reveal fungal community in roots of the turf-grass *Zoysiajaponica*. A total of 317 OTUs were identified from root samples of *Z.japonica* by analysis of ITS amplicon reads. Fungal community was dominated by *Sordariales* (32.45%), followed by *Chaetothyriales* (18.16%), unknown taxa in *Sordariomycetes* (14.63%) and *Pleosporales* (12.48%). During isolation, 151 endophytic fungal strains were obtained from roots of *Z.japonica* and a variety of taxa were found by ITS amplification and sequencing. Moreover, 11 endophytic fungal species were further characterised in this study, based on morphological characterisation and multi-loci phylogenetic analysis, including *Niessliadimorphospora*, a newly-recorded species in Korea and 10 novel species (*Dactylariahwasunensis***sp. nov.**, *Lophiostomajeollanense* sp. nov., *Magnaporthiopsiszoysiae***sp. nov.**, *Poaceascomaendophyticum***sp. nov.**, *P.koreanum***sp. nov.**, *P.magnum***sp. nov.**, *P.zoysiiradicicola***sp. nov.**, *Stagonosporaendophytica* sp. nov., *Setophomazoysiae***sp. nov.** and *Pseudorhypophilapoae***sp. nov.**). Antifungal activities of these species were tested against the turf-grass brown patch pathogen *Rhizoctoniasolani* AG2-2(IIIB), with *S.zoysiae* being the best antagonist. In addition, butanol extract from mycelia of *S.zoysiae* strongly inhibited *R.solani* AG2-2(IIIB) *in vitro* and *in planta*. The results of this study expand the biodiversity of endophytic fungi and revealed potential biological resources for future turf-grass management and bioactive compound exploitation.

## ﻿Introduction

Zoysiagrasses (*Zoysia* spp. Willd.) are mat-forming perennial warm-season grasses belonging to the family *Poaceae*, native to the western Pacific Rim and the Indian Ocean (Tothill and Hacker 1983; Soreng et al. 2015). Zoysiagrasses gained popularity amongst growers for their low maintenance inputs and tolerance to multiple biotic and abiotic stresses. A total of 11 species are reported in the genus *Zoysia*, but only *Z.japonica*, *Z.matrella* and *Z.pacifica* and their interspecific hybrids have been widely used as turf-grass including on golf courses, athletic fields, home lawns and other recreational sites (Chandra et al. 2017). *Zoysiajaponica* is one of the most important turf-grass species distributed mainly in Asia, North and South America and Australia (Loch et al. 2017). Due to its popularity, some biotechnologies, such as conventional breeding and molecular transformation have been used to improve *Z.japonica* (Ge et al. 2006; Yang et al. 2023).

Endophytes colonising intra- or intercellular plant tissues play multifaceted roles in plant-microbe interactions, ranging from promotion of plant growth to biocontrol of pathogens and enhancement of biotic/abiotic tolerance (Sridhar 2019; Bharadwaj et al. 2020). Application of endophytes is increasingly envisioned to reduce the use of agrochemicals and lower production costs (Tiwari et al. 2023). In recent years, endophytic fungi have attracted great attention from researchers and are increasingly being investigated for their ability to secrete various bioactive compounds (secondary metabolites), simulating plant response to environmental stresses, facilitating nutrient uptake and acting as biocontrol agents (Poveda et al. 2020, 2021; Jha et al. 2023). For example, endophytic *Trichoderma* spp. are well known to enhance hosts’ tolerance to multiple stresses and antagonise the growth of a wide range of plant pathogens (Ripa et al. 2019; Rajani et al. 2021). Xia et al. (2019) reported that endophytic fungal species *Aspergillusnidulans*, *Coniothyriumaleuritis*, *Fusariumoxysporum*, *Fusariumproliferatum*, *Pichiaguilliermondii* and *Trichodermaspirale* significantly increased tomato fruit yield. According to Fávaro et al. (2012), the endophyte *Epicoccumnigrum* isolated from sugarcane (*Saccharumofficinarum*) was able to increase root biomass and inhibit several pathogens *in vitro*. In turf-grass, the most frequently studied endophytes belong to the genus *Epichloë* (anamorphs in *Neotyphodium*) (Meyer et al. 2015). *Epichloë* sp. was reported to increase the root growth, metabolic activity and nutrient uptake of ryegrass (*Loliumperenne*), thereby improving its survival in less fertile soil (Chen et al. 2020). It was also found that ryegrass with the presence of the endophyte *Epichloë* showed increased resistance to pathogens *Drechslerasiccans* and *Fusarium* spp. (Wiewióra et al. 2015). Currently, more than 80 commercial turf-grass cultivars contain endophytes and new grass-endophyte combinations are being developed to reduce pesticide use and lower turf maintenance input (Clay 1990; Wiewióra et al. 2015). However, endophytes have not been found in warm-season grasses (Held and Potter 2012).

Although many studies on endophytes have been carried out to capitalise on their potential applications in enhancing agricultural productivity, only a small fraction of endophytes have been isolated and studied and most endophytes remain largely unexplored. Thus, new approaches such as metagenomic and amplicon sequencing have provided more insights into the diversity of endophytes and simplified the analysis of endophytic microbial communities. For fungi, the internal transcribed spacer (ITS) region has been recognised as the standard barcode and either the ITS1 or the ITS2 region is used for high-throughput sequencing to analyse environmental fungal diversity (Monard et al. 2013). Using this technique, Porras-Alfaro et al. (2008) revealed root associated fungi (RAF) in a grass *Boutelouagracilis*, which was dominated by dark septate fungi (DSF) in *Pleosporales*. Khidir et al. (2010) found the presence of RAF in several grass species and a similar cohort of fungal dominants was shared by grasses in semi-arid landscapes. In addition to using a high-throughput sequencing technique alone, an increasing number of studies are incorporating culture-dependent isolation for improved results. For example, Abeywickrama et al. (2023) elucidated fungal community composition associated with *Astrugalussinicus* and *Viciavillosa* by culture-dependent methods and culture-independent amplicon analyses, which is meaningful for green manure practices. Manzotti et al. (2020) analysed the fungal root endophytes community structure of tomato with similar integrative methods and revealed pathogenicity of endophytic fungi by isolation and re-inoculation, suggesting that equilibrium and evenness within the microbiome community are essential for plant health.

**Figure 1.**
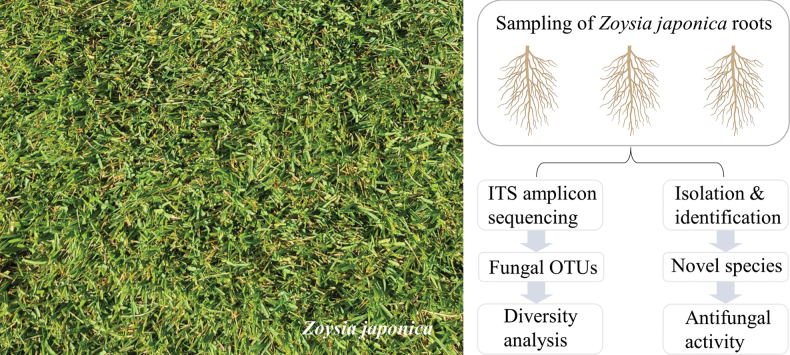
Sampling region of *Zoysiajaponica* from a golf course (left) and schematic diagram of this study (right).

Due to the significance of root-inhabiting endophytic fungi to turf-grasses and because little is known about the endophytes associated with the warm-season turf-grass *Z.japonica*, this study was designed to explore the structure of fungal community in roots of *Z.japonica* through culture-independent and culture-dependent approaches, describe unexplored species and investigate their potential antifungal activities (Fig. 1).

## ﻿Materials and methods

### ﻿Sampling and ITS amplicon sequencing

Healthy plants of *Z.japonica* were sampled from three different sites on a golf course located in Hwasun, South Jeolla Province, Korea. The three samples were washed of soil and the roots of each sample were cut with sterilised scissors. Then the roots were surface disinfected with 2% sodium hypochlorite for 2 min and rinsed three times in sterile distilled water. Genomic DNA from 100 mg of each root sample was extracted using a FavorPrep Plant Genomic DNA Extraction Mini Kit (Favorgen Biotech Corp, Ping-Tung, Taiwan) according to the manufacturer’s instructions. Primer set ITS3 (5’-GCATCGATGAAGAACGCAGC-3’)/ITS4 (5’-TCCTCCGCTTATTGATATGC-3’) was used to amplify the ITS2 region of the internal transcribed spacer. Amplicon sequencing was performed using an Illumina MiSeq platform by the Life is Art of Science (LAS) Laboratory (Gimpo, Korea).

### ﻿Analysis of ITS amplicon sequencing

Quality control of the raw reads from ITS amplicon sequencing of the three *Z.japonica* root samples was performed with FastQC v.0.12.1 (Andrews 2018). Primer sequences in the reads were removed by Cutadapt v.4.0 (Martin 2011). Resulting reads were further processed using a Vsearch v.2.22.1 pipeline (Rognes et al. 2016). First, paired-end reads were merged, truncated to an equal length (300 bp) and quality filtered. Chimeras were removed after *de novo* detection, followed by removal of singletons. Reads were subsequently clustered into operational taxonomic units (OTUs) with a threshold of 0.97 after full-length dereplication. Taxonomic annotation of the OTUs was conducted using a SINTAX algorithm (Edgar 2016) against the ITS reference database UNITE (Nilsson et al. 2019). OTUs annotated to *Zoysia* sp. were removed and only fungal OTUs were retained for data analysis.

The abundance distribution of fungal OTUs in the three root samples was summarised by alpha diversity (within-sample diversity) metrics including Shannon and Simpson indices. Composition of the fungal community in the samples was plotted, based on the relative abundance of OTUs at different taxonomic levels. Dominant fungal OTUs in each sample were identified by analysing the OTUs with relative abundance in the top 20 at the order and genus levels. Sequences of these OTUs were extracted and phylogenetic trees were constructed using the Maximum Likelihood method. The above data analysis and visualisation were performed using the packages vegan v.2.6-4 (Oksanen et al. 2022), phyloseq v.1.42.0 (McMurdie and Holmes 2013) and ggplot2 (Wickham 2011) in R v.4.2.2 software (R Core Team 2019).

### ﻿Fungal isolation

The remaining root samples of *Z.japonica* were used for fungal isolation. Briefly, surface-disinfected roots were cut into small pieces (5 mm long) and placed on potato dextrose agar (PDA) amended with 50 μg ml^-1^ of ampicillin and kanamycin. Plates were incubated in darkness at 25 °C for 3–7 days. Fungal colonies developed from plant tissues were sub-cultured on fresh PDA to obtain pure cultures. All the fungal strains were maintained in the Molecular Microbiology Lab., Department of Integrative Food, Bioscience and Biotechnology, Chonnam National University, Gwangju, Republic of Korea.

### ﻿Fungal DNA extraction and polymerase chain reaction

Fungal genomic DNA was extracted by a modified CTAB method (Cubero et al. 1999) using fresh mycelia grown on PDA. Amplification of gene fragments of ITS, SSU, LSU, *TEF1*, *RPB1*, *RPB2*, *TUB2* and *MCM7* was performed using primer pairs of ITS4/ITS5 (White et al. 1990), NS1/NS4 (White et al. 1990), LR0R/LR5 (Vilgalys and Hester 1990), EF1-983F/EF1-2218R (Rehner and Buckley 2005), RPB1-Ac/RPB1-Cr (Stiller and Hall 1997; Matheny et al. 2002), RPB2-5f/RPB2-7cR (Liu et al. 1999), T1/Bt2b (Glass and Donaldson 1995; O’Donnell and Cigelnik 1997) and MCM7-709/MCM7-1348 (Schmitt et al. 2009), respectively. The PCR products were purified with ExoSap-IT reagent (GE Healthcare, USA) before paired-end Sanger sequencing by Macrogen Inc. (Seoul, Korea).

### ﻿Phylogenetic analysis

The resulting sequences of fungal strains in this study were subjected to BLASTn searches in the NCBI database (https://blast.ncbi.nlm.nih.gov/Blast.cgi) with an additional option (sequences from type materials). Related reference sequences of different genera were accessed, based on recent relative publications (Cai et al. 2006; Zhang et al. 2011, 2023; Quaedvlieg et al. 2013; Luo et al. 2016; Thambugala 2017; Liu et al. 2019; Marin-Felix et al. 2020; Andreasen et al. 2021; Crous et al. 2022a; Wong et al. 2022; Tanney et al. 2023). Accession numbers of the reference strains were shown in Suppl. material 1. A newly-developed tool OFPT (One-click Fungal Phylogenetic Tool) by Zeng et al. (2023) was used for phylogenetic analysis in this study. Complete procedures including sequence retrieving, multi-sequence alignment, sequence trimming, sequence concatenating, model testing and construction of Maximum Likelihood (ML) and Bayesian trees were accomplished by this tool. Briefly, nucleotide substitution models of the datasets were tested by ModelFinder (Kalyaanamoorthy et al. 2017). For the ML analysis, 1,000 bootstrap replicates were performed by IQ-TREE (Nguyen et al. 2015) with the best-fit substitution model. For the Baysian method, Markov Chain Monte Carlo (MCMC) analysis was performed by MrBayes 3.2.7 (Ronquist et al. 2012) with parameters of 50,000,000 generations and a sampling frequency of every 100^th^ generation. The first 25% of the samples were discarded during the calculation of convergence diagnostics. Consensus BI trees with posterior probabilities (PP) were generated after the standard deviation of the runs fell below 0.01. Sequence alignments of phylogenetic analyses were shown in Suppl. material 2.

### ﻿Morphological characterisation

Colonial characteristics of the fungal strains were recorded after incubation on PDA at 25 °C in darkness for 7 days. To stimulate sporulation, fungal strains were inoculated on malt extract agar (MEA) and oatmeal agar (OA) and incubated at 25 °C. Colonies of strains that had not sporulated after 7 days were scratched and exposed to UV light for 2 h and again incubated at 25 °C for further observation. Conidial morphology was observed under an optical microscope (Olympus, Tokyo, Japan) equipped with a differential interference contrast (DIC) module.

### ﻿*In vitro* dual culture assay

Antifungal activity of fungal strains against turf-grass brown patch pathogen *Rhizoctoniasolani* AG2-2(IIIB) KACC 40151 was tested by *in vitro* dual culture assay. Mycelia plugs (5 mm diameter) of endophytic fungi and pathogen *R.solani* AG2-2(IIIB) were placed on PDA (3 cm apart). PDA plates inoculated with only pathogen were used as control. The experiment was repeated twice. Mycelial growth of colonies of pathogens with (a) and without (b) endophytic fungi was measured after incubation at 25 °C for 2 d. Inhibition rate of mycelia growth was calculated as follows: Inhibition rate (%) = 100 − (a/b × 100). Endophytic fungus with the highest inhibition rate was chosen for further investigation of antifungal activity against pathogens *R.cerealis* KACC 40154, *R.solani* AG2-2(IV) KACC 40132, *Clarireediajacksonii* CMML 20-31, *Pythiumultimum* KACC 40705, *Sclerotiniasclerotiorum* KACC 40457, *Botrytiscinerea* CMML 20-BC04, *Fusariumoxysporum* CMML 21-1 and *Colletotrichumgloeosporiodes* KACC 40003.

### ﻿Crude extraction of fungal metabolites

Mycelia of the selected endophytic fungus grown in potato dextrose broth (PDB) for 2 weeks were collected and used as raw material for metabolite extraction. First, a mycelial sample (2 g) was mixed with 8 ml of different solvents (methanol, ethyl acetate, hexane, acetone and butanol) and incubated in a shaker at room temperature for 24 h. Crude extracts were then obtained after centrifugation. Subsequently, antifungal activity against *R.solani* AG2-2(IIIB) was tested on each crude extract using the paper disc method. Briefly, agar plugs (5 mm) of *R.solani* AG2-2(IIIB) were inoculated on the centre of PDA plates, sterile paper discs were then loaded with 20 μl of the crude extracts, air-dried thoroughly and placed on the surface of pathogen-inoculated PDA plates. Mycelial growth inhibition was observed after incubation at 25 °C for 2 d. The experiment was performed three times.

### ﻿Mycelial viability

To test the viability of pathogen *R.solani* AG2-2(IIIB) with or without crude extracts of the selected endophytic fungus, mycelia were picked, placed on glass slides and stained with neutral red (0.1 μg ml^-1^, DaeJung, Siheung, Korea) or Evans blue (0.5 μg ml^-1^, Alfa Aesar, Haverhill, USA). After incubation for 5 min at room temperature, the mycelia were washed three times with sterile distilled water and examined under the microscope.

### ﻿Pot assay

*In planta* antifungal activity against *R.solani* AG2-2(IIIB) was tested using butanol extract of the selected endophytic strain on creeping bentgrass. Mycelia of *R.solani* AG2-2(IIIB) grown in PDB for 3 d were collected and pulverised for inoculation. Approximately 2-week-old creeping bentgrass grown in pots was used for pathogen treatments. Butanol extract (5 ml) of the fungal strain was evaporated and re-dissolved in the same volume of distilled water, then sprayed on the pathogen treated pots. Distilled water was treated as a negative control and the same volume of the fungicide azoxystrobin (20 μg ml^-1^) was treated as a positive control. Three pots were used for each treatment. All the pots were placed in a greenhouse at 25 °C with a light period of 16 h per day until disease was noted.

### ﻿Statistical analysis

Mycelial growth of pathogens in dual culture assays was measured to calculate mycelial growth inhibition rates. Data were used for multiple comparison by the least significant difference (LSD) test (P ≤ 0. 05) in R software (R Core Team 2019).

## ﻿Results

### ﻿ITS amplicon analysis

**Figure 2.**
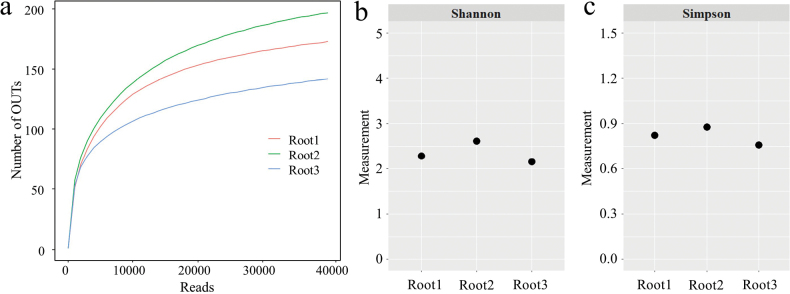
Alpha diversity of fungal communities in *Zoysiajaponica* root samples **a** rarefaction curves for OTUs at different sequence reads **b** measurements of Shannon indices **c** measurements of Simpson indices.

A total of 317 fungal OTUs were obtained from *Z.japonica* root samples, based on amplicon sequencing of the ITS2 region. Sample size-based rarefaction curves show a saturated trend (Fig. 2a), indicating that the community diversity was adequately captured in the three samples. Alpha diversity measurements were similar amongst the samples, ranging from 2.16–2.61 and 0.76–0.88 for Shannon and Simpson indices, respectively (Fig. 2b, c).

**Figure 3.**
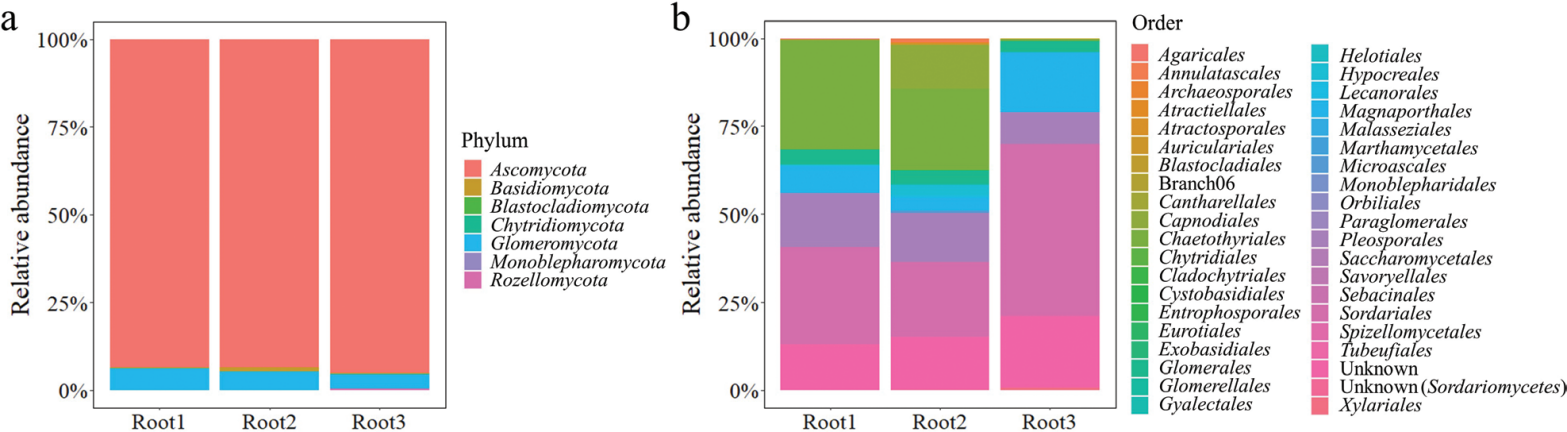
Fungal communities in *Zoysiajaponica* root samples **a** relative abundance at phylum level **b** relative abundance at order level.

Fungal OTUs from root samples of *Z.japonica* were classified into seven phyla, including *Ascomycota*, *Basidiomycota*, *Blastocladiomycota*, *Chytridiomycota*, *Glomeromycota*, *Monoblepharomycota* and *Rozellomycota*, with *Ascomycota* being the most abundant (94.15% on average) phylum (Fig. 3a). A total of 40 orders were found with different relative abundance, including *Agaricales*, *Annulatascales*, *Archaeosporales*, *Atractiellales*, *Atractosporales*, *Auriculariales*, *Blastocladiales*, Branch06 (unclassified order in *Sordariomycetes*), *Cantharellales*, *Capnodiales*, *Chaetothyriales*, *Chytridiales*, *Cladochytriales*, *Cystobasidiales*, *Entrophosporales*, *Eurotiales*, *Exobasidiales*, *Glomerales*, *Glomerallales*, *Gyalectales*, *Helotiales*, *Hypocreales*, *Lecanorales*, *Magnaporthales*, *Malasseziales*, *Marthamycetales*, *Microascales*, *Monoblepharidales*, *Orbiliales*, *Paraglomerales*, *Pleosporales*, *Saccharomycetales*, *Savoryellales*, *Sebacinales*, *Sordariales*, *Spizellomycetales*, *Tubeufiales*, *Xylariales* and two unknown orders (Fig. 3b).

**Figure 4.**
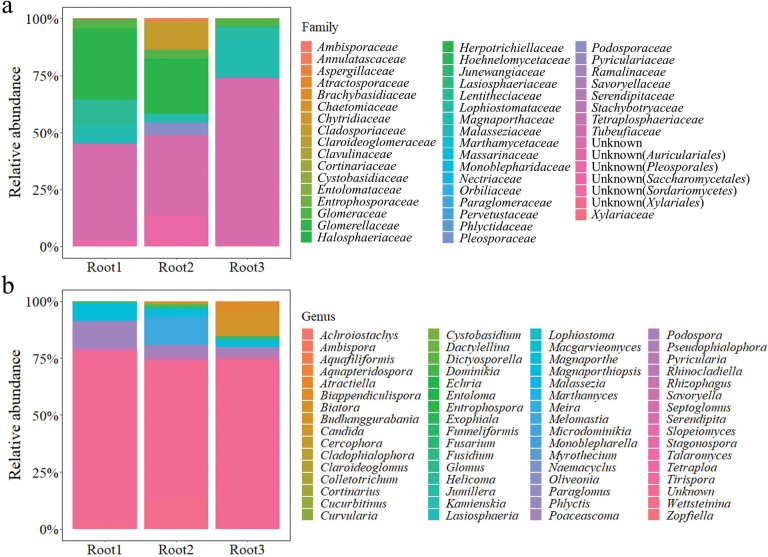
Fungal communities in *Zoysiajaponica* root samples **a** relative abundance at family level **b** relative abundance at genus level.

The composition of OTUs were similar in the three samples at the family and genus levels. A total of 49 families and 64 genera were found in the OTUs and unknown taxa in *Sordariales* dominated the fungal community at both the family and genus levels (Fig. 4). Other classified genera of the OTUs were *Ambispora*, *Atractiella*, *Biatora*, *Budhanggurabania*, *Cladophialophora*, *Colletotrichum*, *Cortinarius*, *Cucurbitinus*, *Curvularia*, *Dictyosporella*, *Dominikia*, *Entrophospora*, *Exophiala*, *Funneliformis*, *Fusarium*, *Fusidium*, *Glomus*, *Helicoma*, *Kamienskia*, *Lasiosphaeria*, *Lophiostoma*, *Macgarvieomyces*, *Magnaporthe*, *Magnaporthiopsis*, *Marthamyces*, *Melomastia*, *Microdominikia*, *Myrothecium*, *Naemacyclus*, *Paraglomus*, *Oliveonia*, *Phlyctis*, *Poaceascoma*, *Podospora*, *Pseudophialophora*, *Rhizophagus*, *Septoglomus*, *Serendipita*, *Slopeiomyces*, *Stagonospora*, *Tetraploa*, *Tirispora* and *Wettsteinina*.

**Figure 5.**
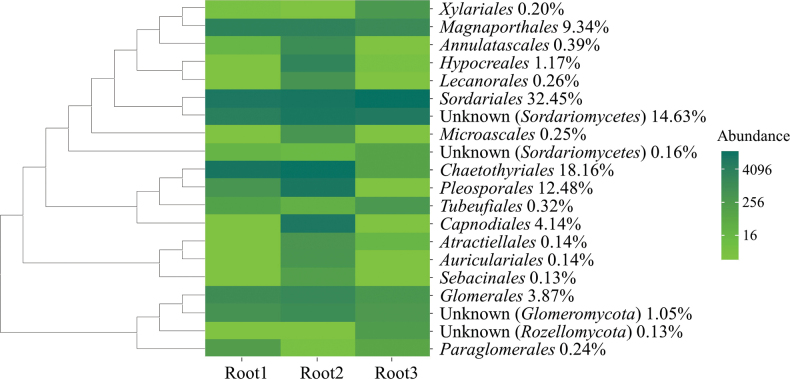
Top 20 abundance orders of fungal communities in *Zoysiajaponica* root samples with their phylogenetic relationships.

In the fungal OTUs, the top 20 relative abundance orders were *Sordariales* (32.45%), *Chaetothyriales* (18.16%), unknown order in *Sordariomycetes* (14.63%), *Pleosporales* (12.48%), *Magnaporthales* (9.34%), *Capnodiales* (4.14%), *Glomerales* (3.87%), *Hypocreales* (1.17%), unknown order in *Glomeromycota* (1.05%), *Annulatascales* (0.39%), *Tubeufiales* (0.32%), *Lecanorales* (0.26%), *Microascales* (0.25%), *Paraglomerales* (0.24%), *Xylariales* (0.20%), unknown order in *Sordariomycetes* (0.16%), *Auriculariales* (0.14%), *Atractiellales* (0.14%), unknown order in *Sebacinales* (0.13%) and unknown order in *Rozellomycota* (0.13%, Fig. 5).

**Figure 6.**
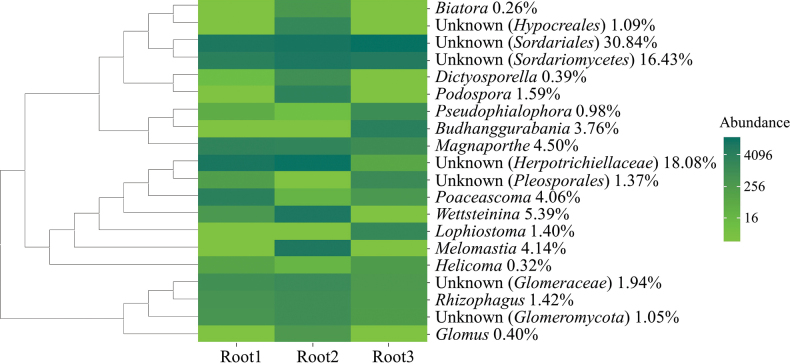
Top 20 abundance genera of fungal communities in *Zoysiajaponica* root samples with their phylogenetic relationships.

Highest relative abundance was found in unknown genera in *Sordariales* (30.84%), followed by unknown genera in *Herpotrichiellaceae* (18.08%) and *Sordariomycetes* (14.63%). The remaining genera in the top 20 in relative abundance were *Wettsteinina* (5.39%), *Magnaporthe* (4.50%), *Melomastia* (4.14%), *Poaceascoma* (4.06%), *Budhanggurabania* (3.76%), unknown genus in *Glome­raceae* (1.94%), *Podospora* (1.59%), *Rhizophagus* (1.42%), *Lophiostoma* (1.40%), unknown genus in *Pleosporales* (1.37%), unknown genus in *Hypocreales* (1.09%), unknown genus in *Glomeromycota* (1.05%), *Pseudophialophora* (0.98%), *Glomus* (0.4%), *Dictyosporella* (0.39%), *Helicoma* (0.32) and *Biatora* (0.26%, Fig. 6).

Raw reads of ITS amplicon sequencing data in this study were deposited into the sequence read archive (SRA) in NCBI with BioProject number PRJNA1165193.

### ﻿Diversity of culturable endophytic fungi

**Figure 7.**
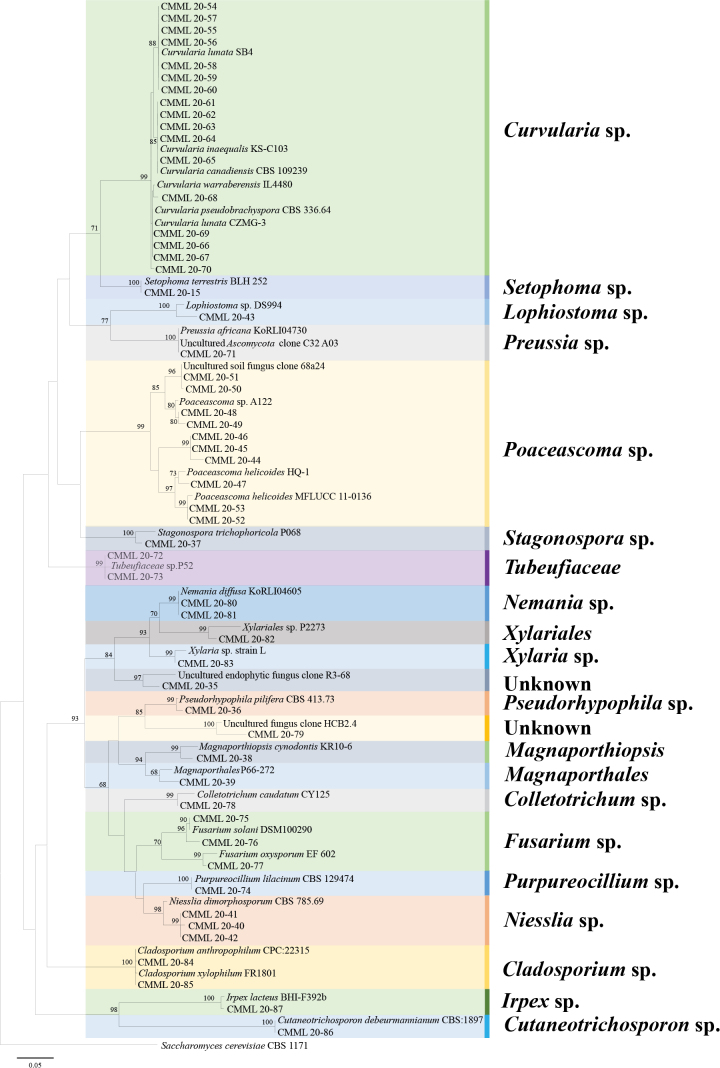
Phylogenetic analysis of fungal strains isolated from *Zoysiajaponica* root samples, based on ITS sequences using Maximum Likelihood method. Bootstrap values (BS) are given at the nodes. *Saccharomycescerevisiae* (CBS 1171) was used as the outgroup taxon.

A total of 151 fungal strains were isolated from roots of *Z.japonica*. Based on colony morphology on PDA, 54 strains with different colony morphology (shape, colour, texture etc.) were preliminarily selected for ITS amplification and sequencing. Resulting sequences of these strains were then used for BLASTn search against NCBI database to obtain reference sequences with high similarities. Phylogenetic analysis revealed that these strains belonged to genera *Curvularia*, *Setophoma*, *Poaceascoma*, *Preussia*, *Lophiostoma*, *Stagonospora*, *Niesslia*, *Purpureocillium*, *Fusarium*, *Collectotrichum*, *Pseudorhypophila*, *Magnaporthiopsis*, *Nemania*, *Xylaria*, *Cladosporium*, *Cutaneotrichosporon*, *Irpex*, genera in *Tubeufiaceae*, *Magnaporthales* and two unknown taxa. (Fig. 7). Amongst these strains, 17 strains (31.48%) were detected as *Curvularia* spp. and 10 strains (18.51%) were identified as *Poaceasoma* spp. Strains with relative low similarities against GenBank were used for separate multi-loci phylogenetic analyses.

### ﻿Taxonomy

#### ﻿ Niesslia dimorphospora

Taxon classificationAnimaliaHypocrealesNiessliaceae

(W. Gams) W. Gams & Stielow (2019).

6A1F04D4-9ABF-5A5E-8F3E-BF99BE44DCD9

Fig. 8

##### Culture characteristics.

**Figure 8.**
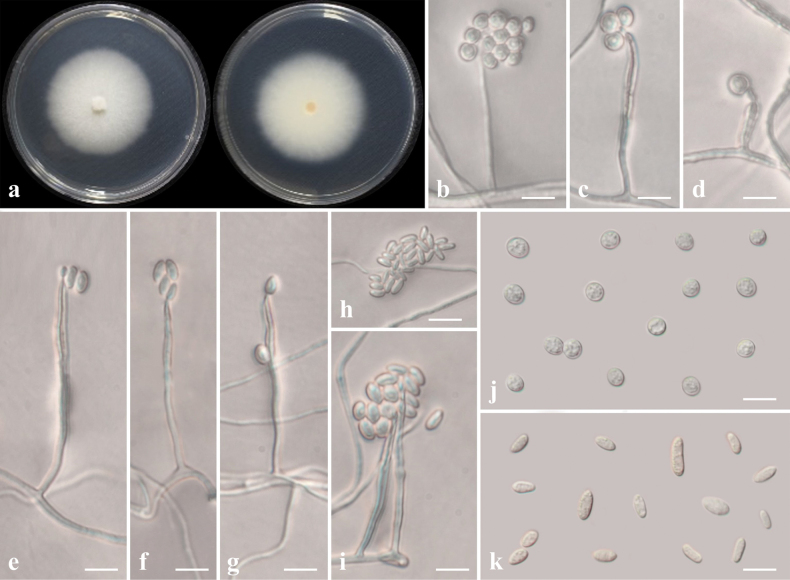
*Niessliadimorphospora* (CMML 20-40) **a** front and reverse sides of colony on PDA**b–d** globose conidia on a conidiophore **e–i** ellipsoidal conidia on a conidiophore **j** globose conidia **k** ellipsoidal conidia. Scale bars: 10 μm.

Colony reaching 53.82 mm diam. after 7 days in darkness at 25 °C on PDA, surface initially floccose, later slimy, white on front and reverse sides (Fig. 8a).

##### Description from living culture CMML 20-40.

Sexual morph: undetermined. Asexual morph: Sporulation abundant on MEA. ***Phialides*** 40–75 μm long, 1.5–2.3 μm wide, thick-walled. ***Conidia*** smooth-walled, globose, 4.5–6.5 μm diam., or ellipsoidal, slightly curved, 6.5–10.5 × 2.2–3.8 μm (Fig. 8b–k).

##### Type.

**Korea** • South Jeolla Province, Hwasun, isolated from roots of *Zoysiajaponica*, October 2020, H. Liu and H. Sang, living cultures CMML 20-40, CMML 20-41 and CMML 20-42.

##### Notes.

**Figure 9.**
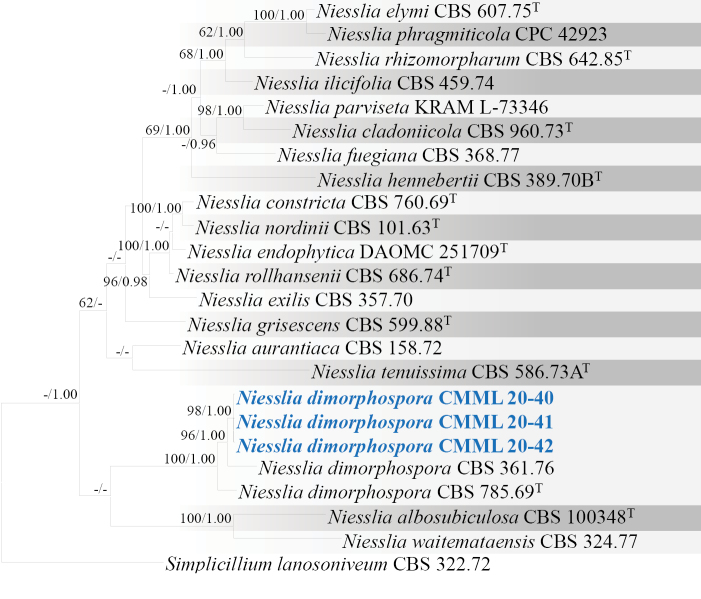
Maximum Likelihood phylogenetic tree, based on combined sequences of ITS, LSU, *RPB2* and *TUB2* from *Niesslia* species. Bootstrap values (BS) and Bayesian posterior probabilities (PP) are given at the nodes (BS/PP). Strains obtained from this study are in bold blue. Ex-type isolates are marked with ^T^. *Simplicilliumlanosoniveum* (CBS 322.72) was used as the outgroup taxon.

*Niessliadimorphospora* typically produce dimorphic conidia (globose and ellipsoidal). In multi-loci phylogenetic analysis using gene sequences of ITS, *TEF1*, *TUB2* and *RPB2*, three strains (CMML 20-40, CMML 20-41 and CMML 20-42) were clustered into a clade containing ex-type strain of *N.dimorphospora* (CBS 785.69) and representative strain CBS 361.76 with high statistical support (100%/1.00) (Fig. 9). This is the first record of *N.dimorphospora* associated with *Z.japonica* in Korea.

#### ﻿ Dactylaria hwasunensis

Taxon classificationAnimaliaHelotiales

H. Liu & H. Sang sp. nov.

E1A98FD6-2D67-558D-AC5B-948087FEE448

857258

Fig. 10

##### Etymology.

Name refers to Hwasun County in Korea, where it was isolated.

##### Description from living culture CMML 20-35.

**Figure 10.**
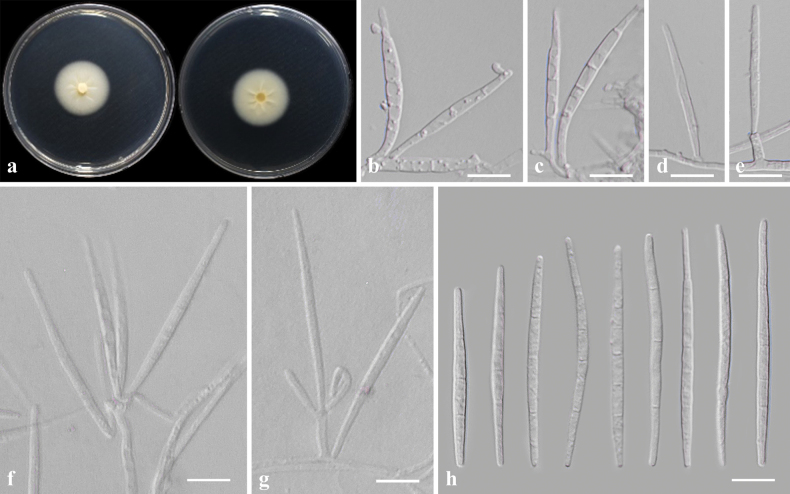
*Dactylariahwasunensis* sp. nov. (CMML 20-35) **a** front and reverse sides of colony on PDA**b–g** conidiophores and conidia **h** conidia. Scale bars: 10 μm.

Sexual morph: undetermined. Asexual morph: Sporulation abundant on MEA. ***Conidiophore*** erect, mironematous to macronematous, aseptate or septate, hyaline, 6–35 μm in length, 2.2–2.8 μm in width. ***Conidiogenous cells*** terminal, integrated, hyaline 2–2.8 μm wide. ***Conidia*** clavate, hyaline, blunt end, 1–5 septate, 10–60 × 2.2–2.8 μm (Fig. 10b–h).

##### Type.

**Korea** • South Jeolla Province, Hwasun, isolated from roots of *Zoysiajaponica*, October 2020, H. Liu and H. Sang, holotype CMML 20-35H (permanently preserved in a metabolically inactive state), ex-holotype CMML 20-35, ex-isotype CMML 20-88.

##### Culture characteristics.

Colony reaching 31.81 mm diam. after 7 days in darkness at 25 °C on PDA, white to yellowish, surface smooth, cracked (Fig. 10a).

##### Notes.

**Figure 11.**
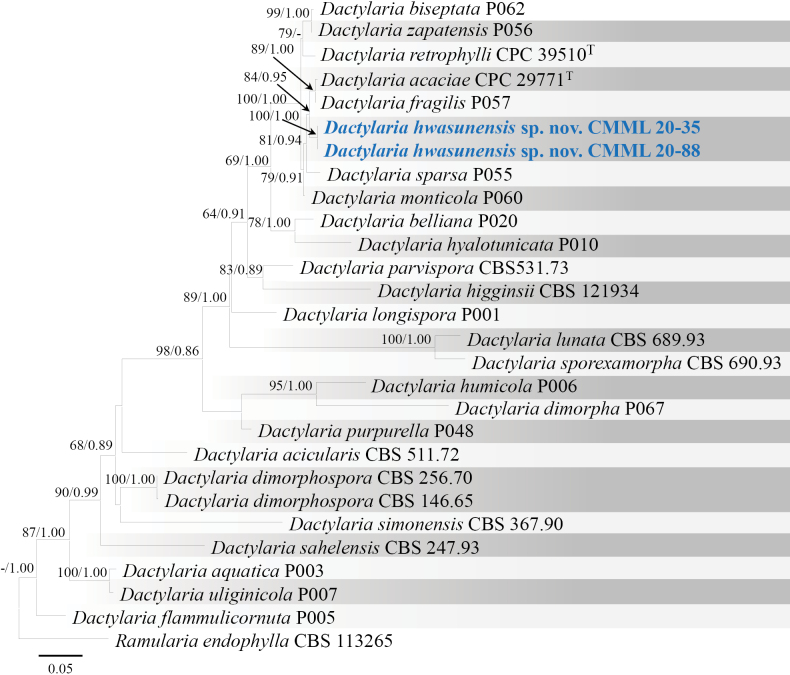
Maximum Likelihood phylogenetic tree, based on LSU sequences from *Dactylaria* species. Bootstrap values (BS) and Bayesian posterior probabilities (PP) are given at the nodes (BS/PP). Strains obtained from this study are in bold blue. Ex-type isolates are marked with ^T^. *Ramulariaendophylla* (CBS 113265) was used as the outgroup taxon.

In phylogenetic analysis of genus *Dactylaria* using sequence data of LSU, the strains used in the present study CMML 20-35 and CMML 20-88 formed a distinct clade sister to clade containing representative strain of *D.fragilis* (P057) and ex-type strain of *D.acaciae* (CPC 29771) with a high statistical support (84%/0.95) (Fig. 11). Based on nucleotide sequences, ex-holotype strain of *D.hwasunensis* (CMML 20-35) differed from ex-type strain of *D.acaciae* (CPC 29771): LSU sequence identities = 806/818 (98.53%). *D.hwasunensis* (CMML 20-35) also differed from representative strain of *D.fragilis* (P057): LSU sequence identities = 844/857 (98.48%). Morphologically, conidial dimensions of *D.hwasunensis* are larger than *D.acaciae* (16–37 × 2–2.5 μm; Crous et al. (2016)) and *D.fragilis* (18–26 × 1.5 µm; De Hoog (1985)). Therefore, *Dactylariahwasunensis* sp. nov. was introduced in this study to accommodate CMML 20-35 and CMML 20-88 in the genus *Dactylaria*.

#### ﻿ Magnaporthiopsis zoysiae

Taxon classificationAnimaliaMagnaporthalesMagnaporthaceae

H. Liu & H. Sang sp. nov.

DF30FF68-95F4-566A-B021-138EBC081D3B

857259

Fig. 12

##### Etymology.

Name refers to its host *Zoysia japonica*.

##### Description from living culture CMML 20-39.

**Figure 12.**
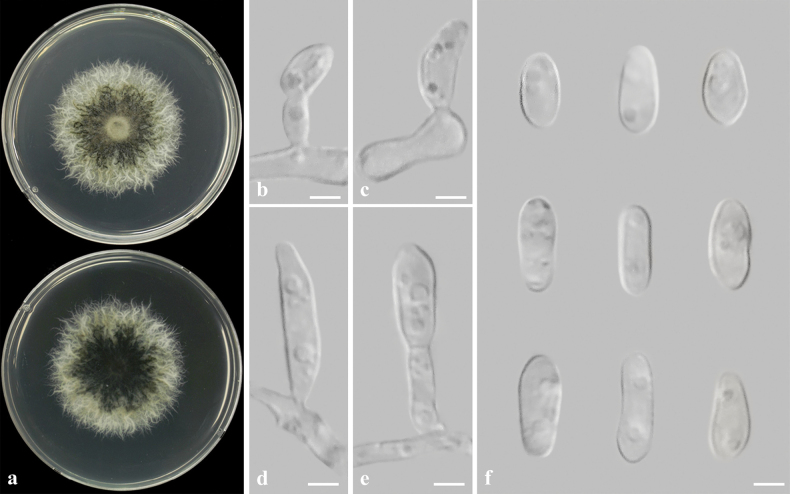
*Magnaporthiopsiszoysiae* sp. nov. (CMML 20-39) **a** front and reverse sides of colony on PDA**b–e** sporulation pattern on OA media **f** conidia. Scale bars: 3 μm.

Sexual morph: undetermined. Asexual morph: Sporulation observed on OA media. ***Conidiophores*** hyaline, single or sometimes branched, septate. ***Conidiogenous cells*** erect or curved, hyaline, 2.5–4 μm in width. ***Conidia*** ovoid or cylindrical, hyaline, slightly curved, 5.5–14.5 × 3.0–5.2 μm (Fig. 12b–h).

##### Type.

**Korea** • South Jeolla Province, Hwasun, isolated from roots of *Zoysiajaponica*, October 2020, H. Liu and H. Sang, holotype CMML 20-39H (permanently preserved in a metabolically inactive state), ex-holotype CMML 20-39, ex-isotype CMML 20-92.

##### Culture characteristics.

Colony reaching 31.81 mm diam. after 7 days in darkness at 25 °C on PDA, centre dark, margin white, mycelia frizzy (Fig. 12a).

##### Notes.

**Figure 13.**
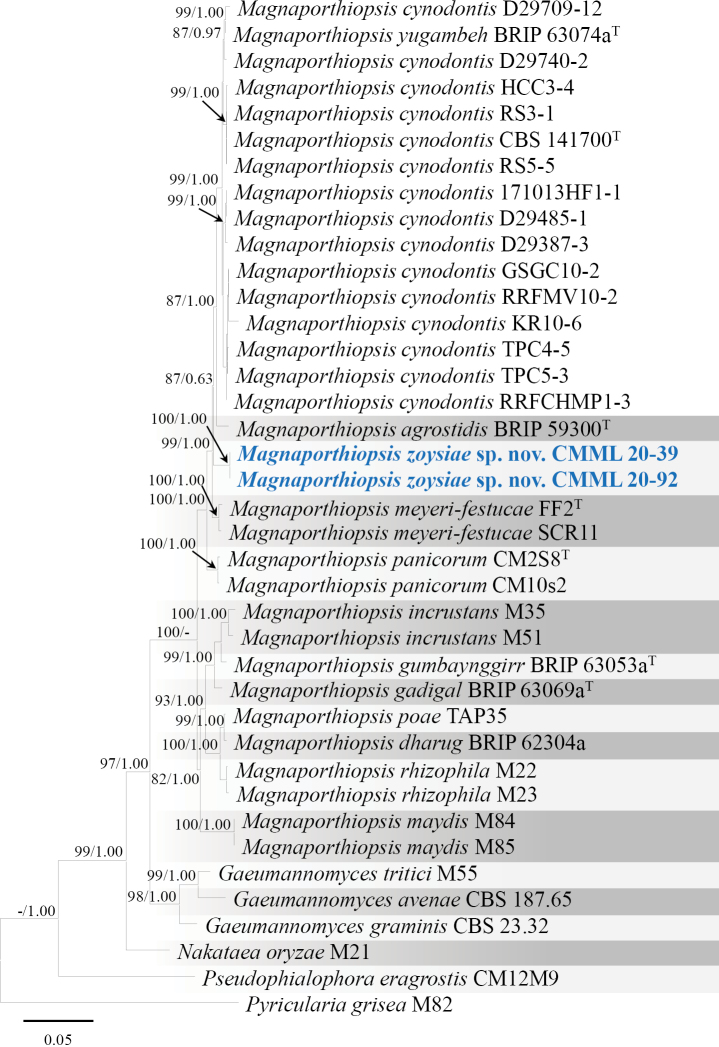
Maximum Likelihood phylogenetic tree, based on combined sequences of ITS, SSU, LSU, *RPB1*, *TEF1* and *MCM7* from *Magnaporthiopsis* species. Bootstrap values (BS) and Bayesian posterior probabilities (PP) are given at the nodes (BS/PP). Strains obtained from this study are in bold blue. Ex-type isolates are marked with ^T^. *Pyriculariagrisea* (M82) was used as the outgroup taxon.

In phylogenetic analysis of *Magnaporthiopsis*, based on sequences of six genes (ITS, SSU, LSU, *RPB1*, *TEF1* and *MCM7*), the strains used in the present study CMML 20-39 and CMML 20-92 fell into a distinct clade with a high statistical support (100%/1.00) (Fig. 13), sister to clades of species *M.cynodontis*, *M.agrostidis* and *M.meyeri-festucae*, which are all turf-grass-associated species. Morphologically, the conidial size of these two strains is larger than those of *M.agrostidis* (4–6 × 1 µm; Crous et al. (2015)) and *M.meyeri-festucae* (3–5 × 1–2.5 µm; Luo et al. (2017)). Conidia of these two strains is slightly longer than *M.cynodontis* (7–13 × 2–6.5 µm; Vines et al. (2020)). Therefore, based on phylogenetic analysis and morphological characteristics, *Magnaporthiopsiszoysiae* sp. nov. was introduced in this study.

#### ﻿ Setophoma zoysiae

Taxon classificationAnimaliaPleosporalesPhaeosphaeriaceae

H. Liu & H. Sang sp. nov.

C5F41A16-9A5D-5E51-BCAC-01BB91D1652C

857260

Fig. 14

##### Etymology.

Name refers to its host genus *Zoysia*.

##### Description from living culture CMML 20-14.

**Figure 14.**
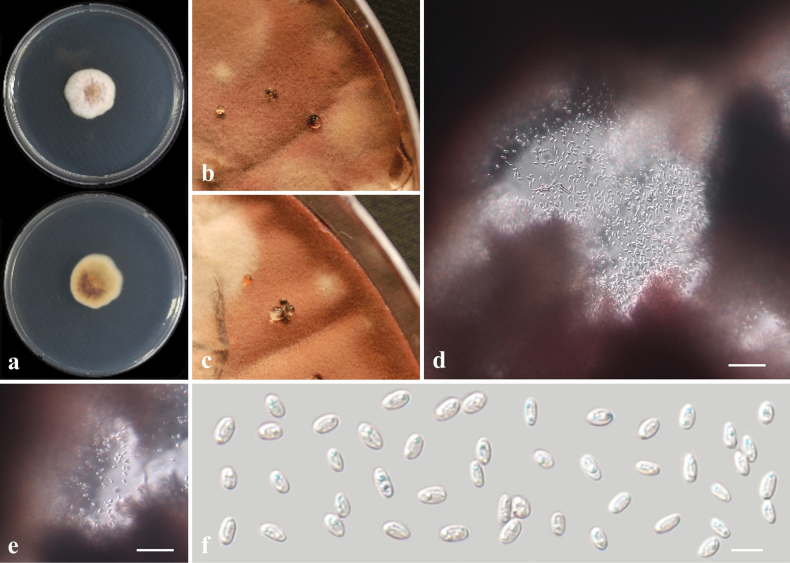
*Setophomazoysiae* sp. nov. (CMML 20-14) **a** front and reverse sides of colony on PDA**b, c** conidiomata on OA media **d–f** conidia. Scale bars: 30 μm (**d, e**); 4.5 μm (**f**).

Sexual morph: undetermined. Asexual morph: Sporulation observed on OA media (Fig. 14b–f). ***Conidiomata*** produced on surface of colonies (Fig. 14b, c). ***Conidia*** ellipsoidal to subcylindrical, aseptate, 3.0–4.6 × 1.8–2.5 μm (Fig. 14d–f).

##### Type.

**Korea** • South Jeolla Province, Hwasun, isolated from roots of *Zoysiajaponica*, October 2020, H. Liu and H. Sang, holotype CMML 20-14H (permanently preserved in a metabolically inactive state), ex-holotype CMML 20-14, ex-isotype CMML 20-15.

##### Culture characteristics.

Colony reaching 28.12 mm diam. in darkness after 7 days at 25 °C on PDA, front side white to light pink, reverse side yellow to sandy brown, mycelia dense (Fig. 14a).

##### Notes.

**Figure 15.**
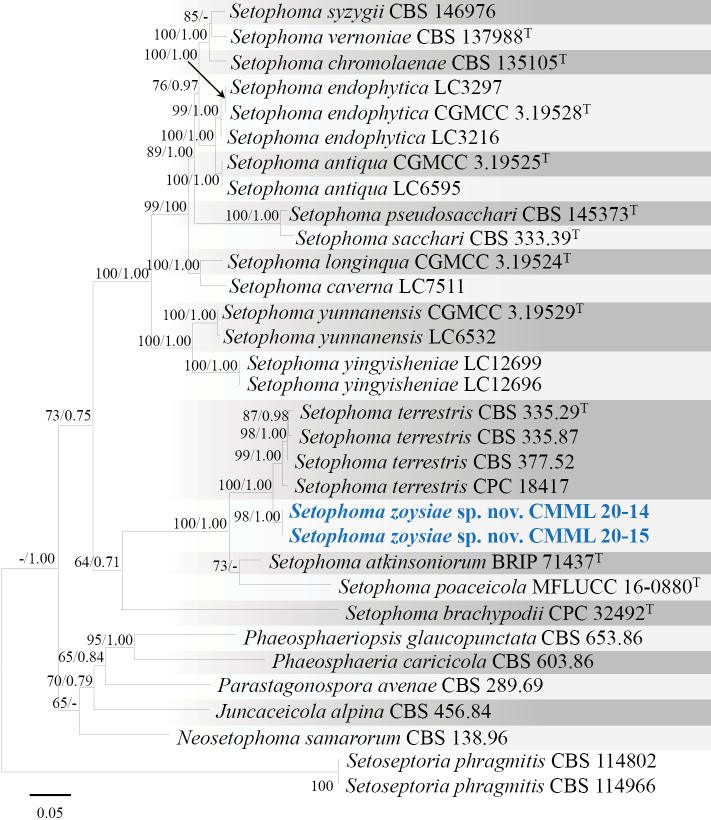
Maximum Likelihood phylogenetic tree based on combined sequences of ITS, LSU, *TEF1*, *RPB2* and *TUB2* from *Setophoma* species. Bootstrap values (BS) and Bayesian posterior probabilities (PP) are given at the nodes (BS/PP). Strains obtained from this study are in bold blue. Ex-type isolates are marked with ^T^. *Setoseptoriaphragmitis* (CBS 114802 and CBS 114966) was used as the outgroup taxon.

Phylogenetic analysis was conducted using dataset from combined sequences of ITS, LSU, *TEF1*, *RPB2* and *TUB2*. The strains CMML 20-14 and CMML 20-15 formed a distinct single branch in the genus *Setophoma*, supported with a high statistical support (100%/1.00) (Fig. 15), sister to clade containing ex-type strain (CBS 335.29) and representative strains (CBS 335.87, CBS 377.52 and CPC 18417) of *S.terrestris*. However, conidia of these two strains are smaller than those of *S.terrestris* (previously *Phomaterrestris*, 4.5–5.5 × 1.8–2.3 μm; Hassen (1929)). Hence, *Setophomazoysiae* sp. nov. was introduced in this study to accommodate CMML 20-14 and CMML 20-15 in the genus *Setophoma*.

#### ﻿ Stagonospora endophytica

Taxon classificationAnimaliaPleosporalesPhaeosphaeriaceae

H. Liu & H. Sang sp. nov.

968069F6-5DA2-5A2C-96B4-E0D7A21CC6E1

857261

Fig. 16

##### Etymology.

Name refers to endophyte.

##### Description from living culture CMML 20-37.

**Figure 16.**
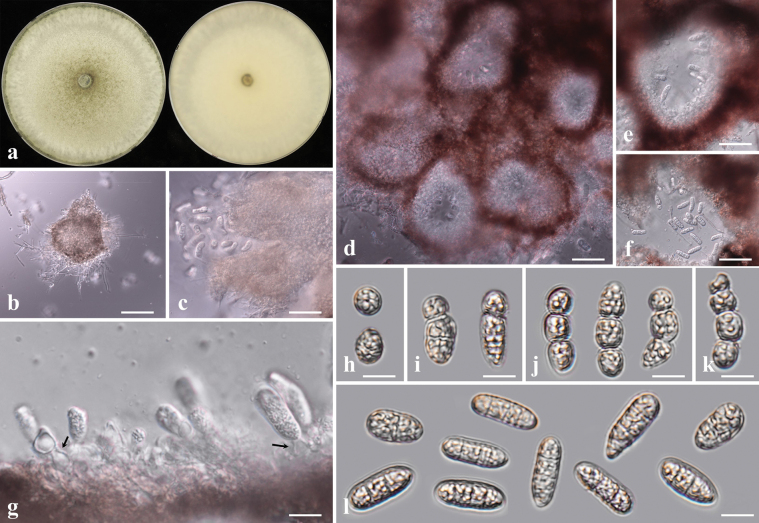
*Stagonosporaendophytica* sp. nov. (CMML 20-37) **a** front and reverse sides of colony on PDA**b, c** conidiomata produced on MEA**d–f** section of conidiomata **g** conidiogenous cell (indicated by arrows) with developing conidia **h–l** conidia. Scale bars: 40 μm (**b–d**); 20 μm (**e**); 50 μm (**f**); 12 μm (**g**); 10 μm (**h–l**).

Sexual morph: undetermined. Asexual morph: Sporulation observed on MEA. ***Conidiomata*** globose, dark brown, 73–105 μm diam. (Fig. 16b–f). ***Conidiophores*** reduced to conidiogenous cells. ***Conidiogenous cells*** 5–8.5 × 4–7.5 μm, hyaline, smooth, ampulliform, produced from the inner wall of conidiomata (Fig. 16g). ***Conidia*** smooth, 1–3 septate, globose or ellipsoidal with obtuse ends, constricted at septa, 15–22 × 7–9 μm (Fig. 16h–l).

##### Type.

**Korea** • South Jeolla Province, Hwasun, isolated from roots of *Zoysiajaponica*, October 2020, H. Liu and H. Sang, holotype CMML 20-37H (permanently preserved in a metabolically inactive state), ex-holotype CMML 20-37, ex-isotype CMML 20-93.

##### Culture characteristics.

Colony reaching the edge of the PDA plates (90 mm) after 7 days in darkness at 25 °C, front side white to yellowish, centre brown, reverse side faint yellow (Fig. 16a).

##### Notes.

**Figure 17.**
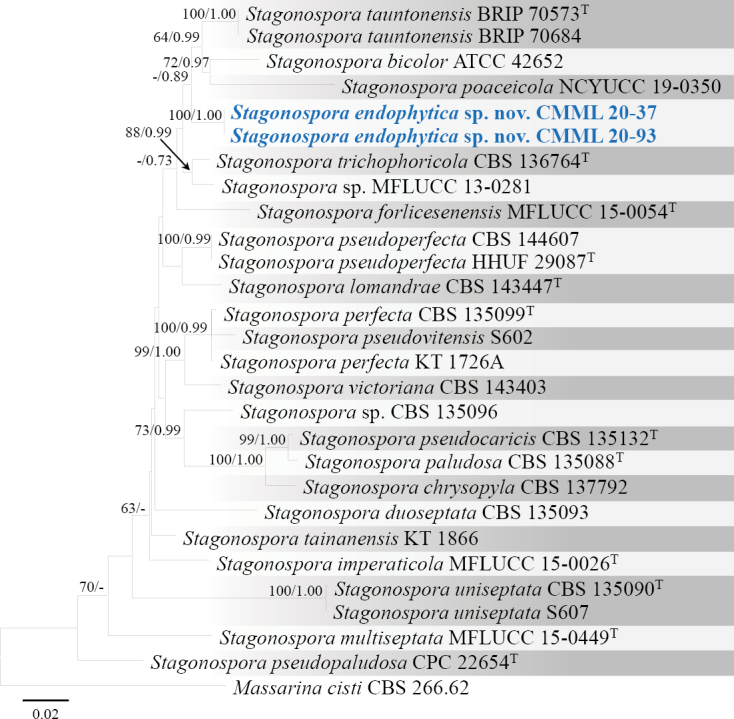
Maximum Likelihood phylogenetic tree, based on combined sequences of ITS, SSU, LSU, *RPB2* and *TUB2* from *Stagonospora* species. Bootstrap values (BS) and Bayesian posterior probabilities (PP) are given at the nodes (BS/PP). Strains obtained from this study are in bold blue. Ex-type isolates are marked with ^T^. *Massarinacisti* (CBS 266.62) was used as the outgroup taxon.

Phylogenetic analysis of *Stagonospora* was performed using sequences of ITS, SSU, LSU, *RPB2* and *TUB2*. Strains in the present study CMML 20-37 and CMML 20-93 fell into a distinct single clade, supported by a high statistical support (100%/1.00) (Fig. 17), sister to clade comprising ex-type strain of *S.tauntonensis* (BRIP 70573) and representative strains of *S.tauntonensis* (BRIP 70684), *S.bicolor* (ATCC 42652) and *S.poaceicola* (NCYUCC 19-0350). In morphology, these two strains differ from *S.tauntonensis* (Crous et al. 2022b) and *S.bicolor* (previously *Leptosphaeriabicolor*; Kaiser et al. (1979)) in having globose conidia and visible contraction at septa of conidia. For *S.poaceicola*, sexual morph was described and asexual morph of this species remains undetermined. Thus, based on phylogenetic analysis and morphological characteristics, *Stagonosporaendophytica* sp. nov. was introduced in this study.

#### ﻿ Pseudorhypophila poae

Taxon classificationAnimaliaSordarialesNaviculisporaceae

H. Liu & H. Sang sp. nov.

9AA30A1B-0636-51A9-BDC7-1FE063C93CF2

857267

Fig. 18

##### Etymology.

Name refers to its host family *Poaceae*.

##### Description from living culture CMML 20-36:

**Figure 18.**
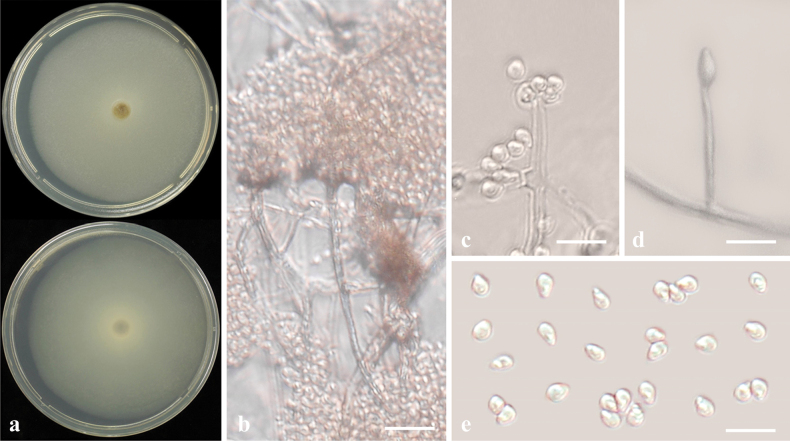
*Pseudorhypophilapoae* sp. nov. (CMML 20-36) **a** front and reverse sides of colony on PDA**b–d** conidiophores and conidia **e** conidia. Scale bars: 10 μm (**b–d**); 8 μm (**e**).

Sexual morph: undetermined. Asexual morph: Sporulation abundant on MEA. ***Conidiophore*** erect, 1.5–2.5 μm in width, ***Conidia*** solitary or in clusters, pyriform, obovoid or triangular, 4.2–5.6 × 2.5–4.5 μm (Fig. 18b–e).

##### Type.

**Korea** • South Jeolla Province, Hwasun, isolated from roots of *Zoysiajaponica*, October 2020, H. Liu and H. Sang, holotype CMML 20-36H (permanently preserved in a metabolically inactive state), ex-holotype CMML 20-36, ex-isotype CMML 20-89.

##### Culture characteristics.

Colony reaching 82.88 mm diam. on PDA after 7 days in darkness at 25 °C, white to buff in both front and reverse sides (Fig. 18a).

##### Notes.

**Figure 19.**
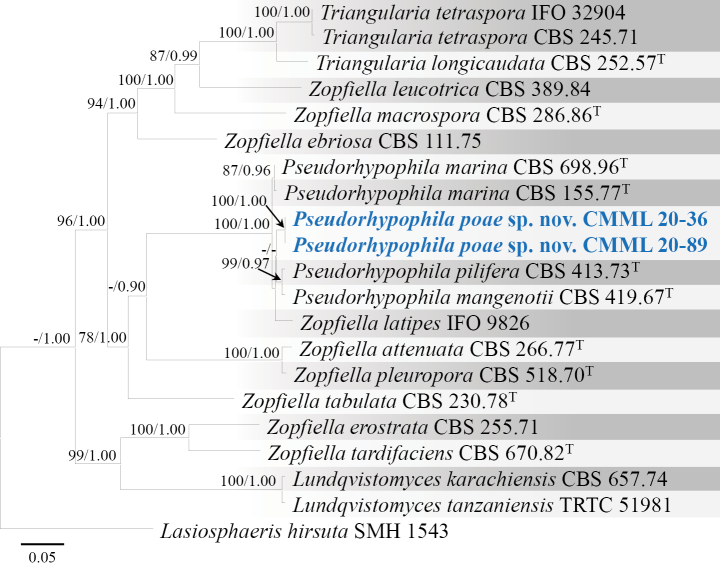
Maximum Likelihood phylogenetic tree based on combined sequences of ITS, LSU, *RPB2* and *TUB2* from *Pseudorhypophila* and relevant genera. Bootstrap values (BS) and Bayesian posterior probabilities (PP) are given at the nodes (BS/PP). Strains obtained from this study are in bold blue. Ex-type isolates are marked with ^T^. *Lasiosphaerishirsuta* (SMH 1543) was used as the outgroup taxon.

The genus *Pseudorhypophila* was recently introduced by accommodating four species including *Triangulariamangenotii*, *Zopfiellamarina*, *Z.pilifera* and *Z.submersa* (Harms et al. 2021). In phylogenetic analysis using combined sequences of ITS, LSU, *RPB2* and *TUB2*, strains in the present study CMML 20-36 and CMML 20-89 formed a single clade in the genus *Pseudorhypophila* supported with a high statistical support (100%/1.00) close to clade comprising ex-type strains of *P.pilifera* (CBS 413.73) and *P.mangenotii* (CBS 419.67) (Fig. 19). However, both *P.pilifera* and *P.mangenotii* produce sexual morph, which was not observed in strains CMML 20-36 and CMML 20-89. In addition, these two strains differ from *P.mangenotii* in producing conidia singly or in clusters, whereas the latter produces conidia singly (Harms et al. 2021). Therefore, based on phylogenetic analysis and morphological characteristics, *Pseudorhypophilapoae* sp. nov. was introduced in this study.

#### ﻿ Lophiostoma jeollanense

Taxon classificationAnimaliaPleosporalesLophiostomataceae

H. Liu & H. Sang sp. nov.

8872B226-F724-5AC1-9478-4CA904F41571

857262

Fig. 20

##### Etymology.

Name refers to Jeolla Province in Korea, the place it was isolated from.

##### Description.

*Lophiostomajeollanense* differs from its closest phylogenetic neighbour, *L.japonicum* (KT573) by unique fixed alleles in three loci: ITS positions 25 (A), 26 (G), 31 (indel), 40 (indel), 70 (C), 91 (C), 93 (G), 114 (G), 132 (T), 134 (A), 136 (C), 138 (T), 142 (G), 364 (G), 365 (A), 368 (T), 383 (T), 407 (C); LSU positions 41 (T), 43 (C), 155 (T), 614 (C); *TEF1* positions 42 (C), 127 (T), 128 (C), 129 (C), 162 (T), 222 (C), 225 (C), 240 (T), 249 (T), 318 (T), 336 (C), 342 (C), 351 (T), 372 (C), 399 (T), 405 (C), 408 (T), 442 (G), 465 (C), 477 (G), 492 (C), 528 (C), 537 (C), 663 (T), 669 (C), 672 (G), 693 (C), 705 (C), 708 (T), 735 (T), 748 (G), 780 (G), 792 (C).

##### Type.

**Korea** • South Jeolla Province, Hwasun, isolated from roots of *Zoysiajaponica*, October 2020, H. Liu and H. Sang, holotype CMML 20-43H (permanently preserved in a metabolically inactive state), ex-holotype CMML 20-43, ex-isotype CMML 20-90.

##### Culture characteristics.

**Figure 20.**
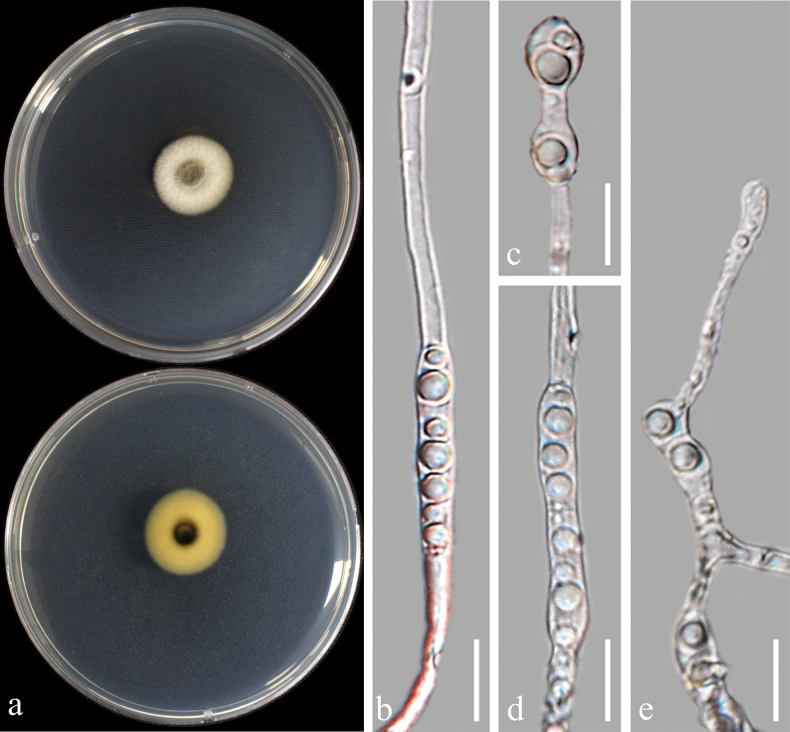
*Lophiostomajeollanense* sp. nov. (CMML 20-43) **a** front and reverse sides of colony on PDA**b–e** chlamydospore-like structures on OA. Scale bars: 10 μm.

Colony reaching 22.24 mm diam. on PDA after 7 days in darkness at 25 °C, surface white to light brown, reverse side yellow, mycelia dense (Fig. 20a).

##### Notes.

**Figure 21.**
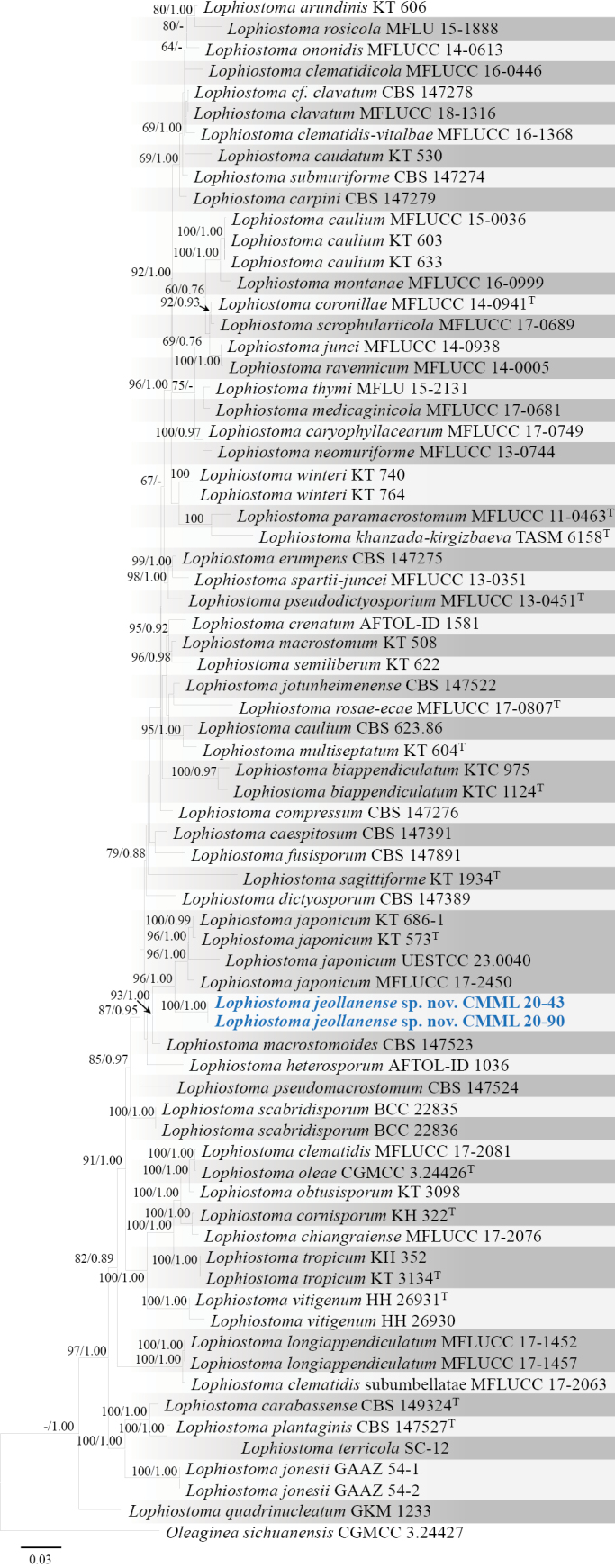
Maximum Likelihood phylogenetic tree based on combined sequences of ITS, LSU, *TEF1* and *RPB2* from *Lophiostoma* species. Bootstrap values (BS) and Bayesian posterior probabilities (PP) are given at the nodes (BS/PP). Strains obtained from this study are in bold blue. Ex-type isolates are marked with ^T^. *Oleagineasichuanensis* (CGMCC 3.24427) was used as the outgroup taxon.

*Lophiostomajeollanense* did not sporulate on synthetic media. Chlamydospore-like structures within mycelia were observed on OA after two weeks (Fig. 20b–e). In phylogenetic analysis of the genus *Lophiostoma*, based on combined sequences of ITS, LSU, *TEF1* and *RPB2*, strains CMML 20-43 and CMML 20-90 formed a distinct single clade with a high statistical support (96%/1.00) (Fig. 21), sister to clade comprising an ex-type strain (KT573) and representative strains (KT 686-1, UESTCC 23.0040 and MFLUCC 17-2450) of *L.japonicum*. Based on nucleotide sequences of three loci, ex-holotype strain of *L.jeollanense* (CMML 20-43) was different from the ex-type strain of *L.japonicum* (KT573): ITS sequence identities = 494/513 (96.30%), gaps = 2; LSU sequence identities = 851/855 (99.53%); TEF sequence identities = 835/868 (96.20%). The species *L.japonicum* (previously *Biappendiculisporajaponica*) was found as a saprophyte on dead stems of unknown herbaceous plants with its sexual morph (Thambugala et al. 2015), whereas strains CMML 20-43 and CMML 20-90 were isolated from roots of *Z.japonica* as a potential endophyte and only chlamydospore-like structures were observed in these strains. Therefore, *Lophiostomajeollanense* sp. nov. was introduced in this study to accommodate CMML 20-43 and CMML 20-90 in the genus *Lophiostoma*.

#### ﻿ Poaceascoma magnum

Taxon classificationAnimaliaPleosporalesLentitheciaceae

H. Liu & H. Sang sp. nov.

A5DA9918-EABF-59E9-9092-3E4502B10D4F

857263

Fig. 22

##### Etymology.

Name refers to the character of large chlamydospores produced by this fungus.

##### Description.

Chlamydospores 10–85 μm in length and 15–23 μm in width, hyaline to dark, clavate, sometimes dumb-bell-shaped or gourd-shaped, straight or sometimes curved. *Poaceascomamagnum* differs from its closest phylogenetic neighbour, *L.lochii* (BRIP 71546) by unique fixed allels in two loci: ITS positions 49 (G), 57 (G), 65 (C), 67 (C), 70 (C), 71 (A), 73 (G), 76 (T), 77 (C), 79 (C), 95 (C), 133 (T), 137 (A), 143 (C), 152 (T), 157 (C), 158 (A), 162 (G), 163 (indels), 169 (A), 184 (C), 190 (T), 192 (G), 194 (A), 376 (indels), 440 (C), 444 (G), 446 (T), 474 (C), 480 (T), 481 (G), 482 (T), 483 (A), 511 (T), 512 (G), 515 (indel), 528 (A), 542 (T), 549 (indel), 560 (T); LSU positions 99 (G), 138 (G), 206 (G), 208 (A), 291 (T), 693 (C), 695 (C), 696 (indel).

##### Culture characteristics.

**Figure 22.**
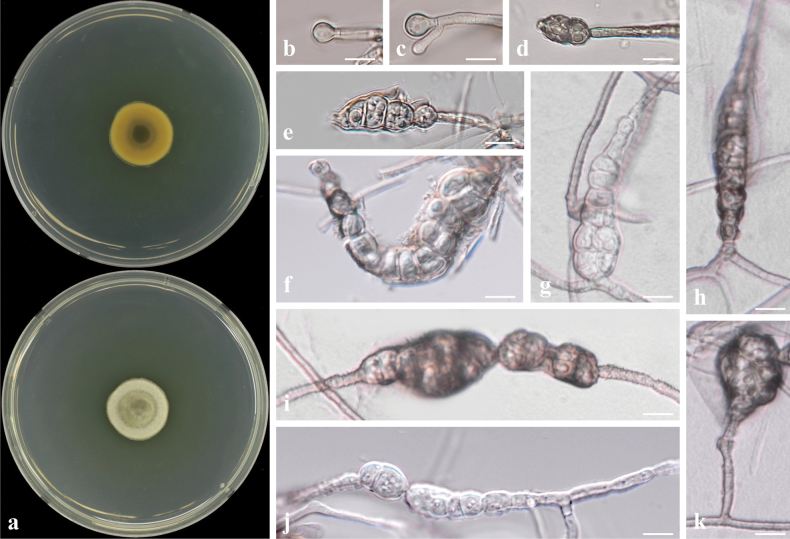
*Poaceascomamagnum* sp. nov. (CMML 20-47) **a** front and reverse sides of colony on PDA**b–k** chlamydospores on MEA. Scale bars: 10 μm.

Colony reaching 22.02 mm diam. on PDA after 7 days in darkness at 25 °C, white to grey at the edge, centre tawny, reverse side yellow brown (Fig. 22a).

##### Type.

**Korea** • South Jeolla Province, Hwasun, isolated from roots of *Zoysiajaponica*, October 2020, H. Liu and H. Sang, holotype CMML 20-47H (permanently preserved in a metabolically inactive state), ex-holotype CMML 20-47, ex-isotype CMML 20-91.

##### Notes.

Sporulation was not observed during culture on synthetic media. On MEA, strains CMML 20-47 and CMML 20-91 produced large (10–85 × 15–23 μm), clavate, hyaline to dark, intercalary or terminal chlamydospores (Fig. 22b–k). Phylogenetic analysis using multi-loci of ITS, LSU, SSU and *TEF1* revealed that strains CMML 20-47 and CMML 20-91 formed a single clade within the genus *Poaceascoma* with a strong statistical support (100%/1.00) basal to clade containing ex-type strains of *P.lochii* (BRIP 71546), *P.helicoides* (MFLUCC 11-0136), *P.herbaceum* (GZCC 19-0046) and representative strain of *P.helicoides* (MFLU 11-0172) (Fig. 26). In comparison of nucleotide sequences of ITS and LSU, ex-holotype strain of *P.magnum* (CMML 20-47) differed from ex-type strain of *P.lochii* (BRIP 71546): ITS identities = 491/526 (93.35%), 38 gaps; LSU identities = 889/896 (99.22%). In addition, *P.magnum* (CMML 20-47) differed from ex-type strain of *P.helicoides* (MFLUCC 11-0136) in four loci: ITS identities = 435/469 (92.75%), 71 gaps; SSU identities = 914/916 (99.78%); LSU identities = 786/794 (98.99%); *TEF1* identities = 879/924 (95.13%). *P.magnum* (CMML 20-47) also differed from ex-type strain of *P.herbaceum* (GZCC 19-0046) in these loci: ITS identities = 390/420 (92.86%), 69 gaps; SSU identities = 1021/1025 (99.61%); LSU identities = 891/900 (99.00%); *TEF1* identities = 883/924 (95.56%). Morphologically, this fungus differs from other *Poaceascoma* spp. by producing large and sometimes dark chlamydospores. Therefore, *Poaceascomamagnum* sp. nov. was introduced in this study to accommodate CMML 20-47 and CMML 20-91.

#### ﻿ Poaceascoma endophyticum

Taxon classificationAnimaliaPleosporalesLentitheciaceae

H. Liu & H. Sang sp. nov.

96A5E393-1F3B-52F1-B51A-654B624FB71E

857264

Fig. 23

##### Etymology.

Name refers to endophyte.

##### Description.

*Poaceascomaendophyticum* differs from its closest phylogenetic neighbour, *P.halophilum* (MFLUCC 15-0949) by unique fixed alleles in two loci: LSU positions 84 (T), 88 (indel), 280 (C), 484 (C), 534 (T), 654 (T), 691 (T), 766 (indel), 800 (indel); SSU position 174 (indel), 972 (indel).

##### Culture characteristics.

**Figure 23.**
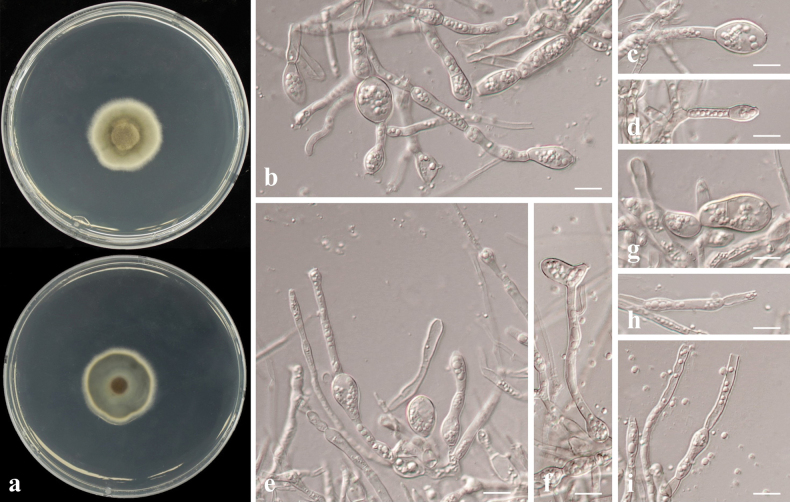
*Poaceascomaendophyticum* sp. nov. (CMML 20-48) **a** front and reverse sides of colony on PDA**b–i** chlamydospore-like structures on MEA. Scale bars: 10 μm.

Colony reaching 29.33 mm diam. on PDA after 7 days in darkness at 25 °C, white ring at the edge, centre brownish, reverse side dark brown with a white edge, mycelia dense (Fig. 23a).

##### Type.

**Korea** • South Jeolla Province, Hwasun, isolated from roots of *Zoysiajaponica*, October 2020, H. Liu and H. Sang, holotype CMML 20-48H (permanently preserved in a metabolically inactive state), ex-holotype CMML 20-48, ex-isotype CMML 20-49.

##### Notes.

Strains CMML 20-48 and CMML 20-49 did not sporulate on synthetic media. Only chlamydospore-like structures were observed on MEA after two weeks, mostly elliptic or oval in shape and 5.5–12.5 μm in width (Fig. 23b–i). In phylogeny, based on multi-loci of ITS, LSU, SSU and *TEF1*, strains CMML 20-48 and CMML 20-49 clustered into a distinct clade, sister to clade comprising ex-type strains of *P.halophilum* (MFLUCC 15-0949), *P.zoysiiradicicola* (CMML 20-50) and representative strain CMML 20-51. Based on nucleotide sequence, ex-holotype strain of *P.endophyticum* (CMML 20-48) differed from ex-type strain of *P.halophilum* (MFLUCC 15-0949) in LSU sequence (identities = 830/836, 99.28%). *P.endophyticum* (CMML 20-48) also differed from *P.zoysiiradicicola* (CMML 20-50 and CMML20-51) in three different loci: ITS identities = 523/558 (93.73%), 40 gaps; LSU identities = 828/838 (98.81%); *TEF1* identities = 824/849 (97.06%). According to (Hyde et al. 2017), colonies of *P.halophilum* on PDA reaches 20–30 mm diameter after 4 weeks, indicating a slower vegetative growth rate than *P.endophyticum*. In addition, *P.endophyticum* differs from *P.zoysiiradicicola* in producing larger chlamydospore-like structures. Thus, *Poaceascomaendophyticum* sp. nov. was introduced in this study to accommodate CMML 20-50 and CMML 20-51 in the genus *Poaceascoma*.

#### ﻿ Poaceascoma koreanum

Taxon classificationAnimaliaPleosporalesLentitheciaceae

H. Liu & H. Sang sp. nov.

9DC8221A-A1A5-54E7-A3B8-5AA4094A9105

857265

Fig. 24

##### Etymology.

Name refers to Korea, the country from where it was isolated.

##### Description.

*Poaceascomakoreanum* differs from its closest phylogenetic neighbour *P.lochii* (BRIP 71546) by unique fixed alleles in two loci: ITS positions 13 (A), 16 (C), 19 (G), 20 (T), 21 (C), 22 (G), 28 (G), 29 (indels), 41 (C), 42 (C), 44 (C), 45 (T), 46 (C), 47 (G), 50 (T), 51 (T), 52 (C), 58 (G), 60 (C), 68 (C), 84 (T), 98 (C), 107 (indel), 109 (C), 112 (indels), 114 (G), 116 (C), 124 (G), 125 (A), 127 (C), 130 (C), 131 (T), 132 (C), 136 (A), 137 (G), 140 (T), 141 (T), 144 (A), 153 (indel), 155 (G), 156 (T), 157 (A), 158 (C), 165 (C), 166 (G), 168 (A), 176 (A), 350 (indels), 388 (C), 391 (T), 397 (G), 404 (T), 410 (A), 420 (C), 435 (C), 440 (C), 443 (G), 447 (C), 449 (G), 450 (A), 469 (T), 475 (G), 476 (T), 481 (T), 489 (T), 495 (A), 497 (G), 500 (A), 502 (C); LSU positions 100 (G), 104 (indel), 138 (C), 141 (G), 143 (G), 145 (G), 205 (C), 206 (C), 210 (C), 488 (C), 550 (T), 700 (C), 705 (C), 755 (G), 907 (T).

##### Culture characteristics.

**Figure 24.**
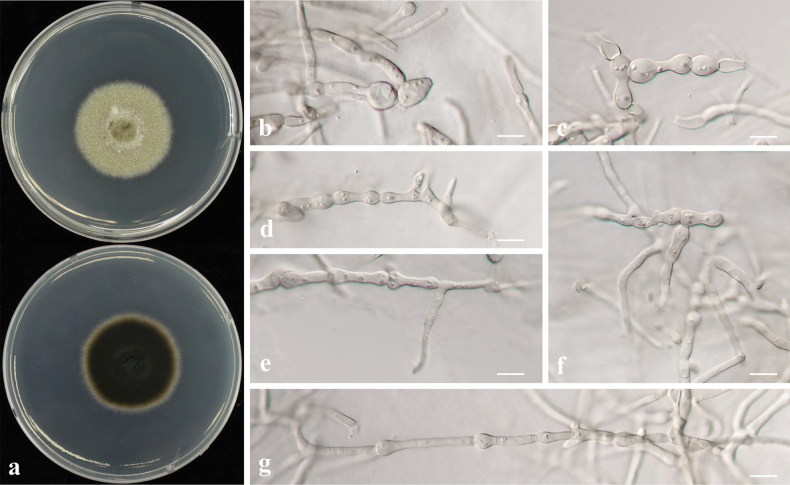
*Poaceascomakoreanum* sp. nov. (CMML 20-44) **a** front and reverse sides of colony on PDA**b–g** chlamydospore-like structures on MEA. Scale bars: 10 μm.

Colony reaching 39.72 mm diam. on PDA after 7 days in darkness at 25 °C, front side greyish-yellow, reverse side black-brown, margins burr-like (Fig. 24a).

##### Type.

**South Korea** • South Jeolla Province, Hwasun, isolated from roots of *Zoysiajaponica*, October 2020, H. Liu and H. Sang, holotype CMML 20-44H (permanently preserved in a metabolically inactive state), ex-holotype CMML 20-44, ex-isotype CMML 20-45; CMML 20-46.

##### Notes.

No conidiogenous structures or sexual morph were observed in strains CMML 20-44, CMML 20-45 and CMML 20-46. On MEA, chlamydospore-like structures (4.5–10.5 μm in width) in hyphae were observed after incubation for two weeks (Fig. 24b–g). In multi-loci phylogeny, based on ITS, LSU, SSU and *TEF1*, these three strains formed a distinct single clade within the genus *Poaceascoma* sister to clade comprising strain of *P.magnum* (ex-type CMML 20-47 and ex-isotype CMML 20-71), *P.herbaceum* (ex-type GZCC 19-0046), *P.helicoides* (ex-type MFLUCC 11-0136 and representative strain MFLU 11-0172) and *P.lochii* (ex-type BRIP 71546) (Fig. 26). Based on nucleotide sequence comparison, *P.koreanum* (CMML 20-44) differed from ex-type strain of *P.lochii* (BRIP 71546) in two loci: ITS identities = 456/518 (88.03%), 40 gaps; LSU identities = 882/896 (98.44%). *P.koreanum* (CMML 20-44) differed from ex-type strain of *P.helicoides* (MFLUCC 11-0136) in four loci: ITS identities = 381/428 (89.02%), 41 gaps; SSU identities = 915/916 (99.89%); LSU identities = 779/793 (98.23%); *TEF1* identities = 896/958 (93.53%). Compared to ex-type of *P.herbaceum* (GZCC 19-0046), nucleotide sequences were different in these loci: ITS identities = 335/380 (88.16%), 41 gaps; SSU identities = 1021/1025 (99.61%); LSU identities = 890/904 (98.45%); *TEF1* identities = 897/958 (93.63%). *P.koreanum* (CMML 20-44) also differed from ex-holotype strain of *P.magnum* (CMML 20-47): ITS identities = 433/500 (89.02%), 45 gaps; SSU identities = 1028/1032 (99.61%); LSU identities = 884/900 (98.22%); *TEF1* identities = 874/924 (94.59%). All of these species were originally found on herbaceous plants. Specifically, *P.lochii* was found on leaves of turf-grass *Zoysiamatrella*, *P.helicoides* and *P.herbaceum* were saprophytes on dead stem of *Digitariasanguinalis* and dead culm of unidentified herbaceous plants, respectively (Phookamsak et al. 2015; Hyde et al. 2017; Tan et al. 2021). Morphologically, *P.herbaceum*, *P.helicoides* and *P.lochii* were described, based on their sexual morph, while only chlamydospore-like structures were observed in strains CMML 20-44, CMML 20-45 and CMML 20-46. Additionally, *P.magnum* differs from these strains in producing large chlamydospores. Therefore, *Poaceascomakoreanum* sp. nov. was introduced in this study.

#### ﻿ Poaceascoma zoysiiradicicola

Taxon classificationAnimaliaPleosporalesLentitheciaceae

H. Liu & H. Sang sp. nov.

750CA9C4-8BAE-5F61-9F46-57798938BF56

857266

Fig. 25

##### Etymology.

Name refers to roots of *Zoysia japonica*.

##### Description.

*Poaceascomazoysiiradicicola* differs from its closest phylogenetic neighbour *P.halophilum* (MFLUCC 15-0949) by unique fixed alleles in two loci: LSU positions 2 (T), 48 (T), 49 (T), 52 (indel), 144 (T), 359 (T), 458 (T), 655 (T), 730 (indel), 764 (indel), 866 (A); SSU positions 171 (indel), 969 (indel).

##### Culture characteristics.

**Figure 25.**
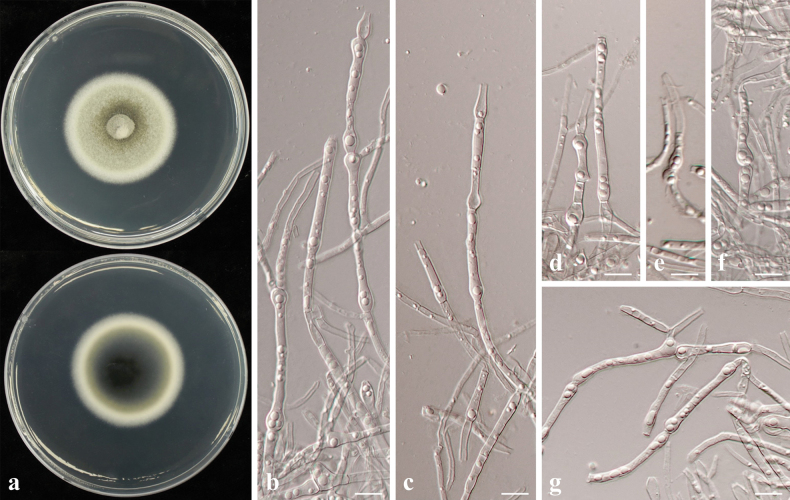
*Poaceascomazoysiiradicicola* sp. nov. (CMML 20-50) **a** front and reverse sides of colony on PDA**b–g** chlamydospore-like structures on MEA. Scale bars: 10 μm.

Colony reaching 44.23 mm diam. on PDA after 7 days in darkness at 25 °C, front side reseda green, reverse sides crineous to dark, margin white on both sides (Fig. 25a).

##### Type.

**Korea** • South Jeolla Province, Hwasun, isolated from roots of *Zoysiajaponica*, October 2020, H. Liu and H. Sang, holotype CMML 20-50H (permanently preserved in a metabolically inactive state), ex-holotype CMML 20-50, ex-isotype CMML 20-51.

##### Notes.

**Figure 26.**
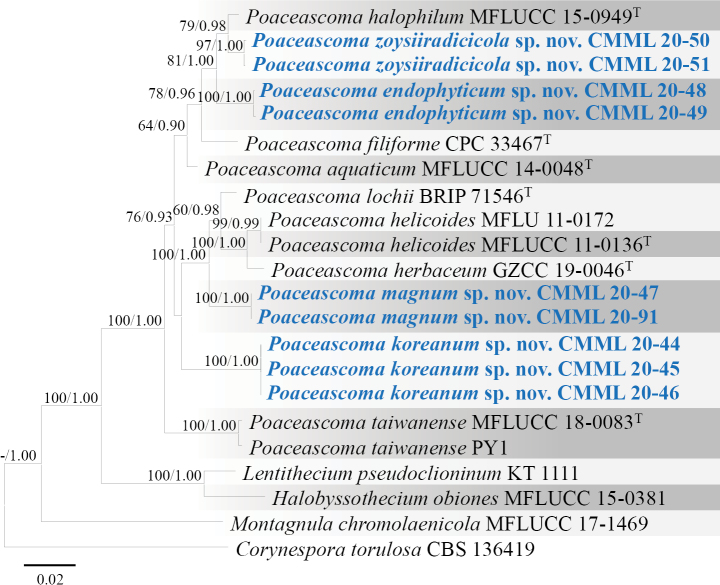
Maximum Likelihood phylogenetic tree based on combined sequences of ITS, LSU, SSU and *TEF1* from *Poaceascoma* species using Maximum Likelihood method. Bootstrap values (BS) and Bayesian posterior probabilities (PP) are given at the nodes (BS/PP). Strains obtained from this study are in bold blue. Ex-type isolates are marked with ^T^. *Corynesporatorulosa* (CBS 136419) was used as the outgroup taxon.

No sporulation was found on synthetic media in this fungus. However, chlamydospore-like structures in hyphae were observed on MEA after two weeks, 4–6.5 μm in width (Fig. 25b–g). In phylogenetic analysis, based on multi-loci of ITS, LSU, SSU and *TEF1*, strains CMML 20-50 and CMML 20-51 formed a separate clade sister to ex-type strain of *P.halophilum* (MFLUCC 15-0949) (Fig. 26). Based on nucleotide sequences of LSU and SSU, ex-holotype strain of *P.zoysiiradicicola* (CMML 20-50) was different with the ex-type strain of *P.halophilum* (MFLUCC 15-0949): LSU sequence identities = 855/863 (99.07%), gaps = 3; SSU sequence identities = 1038/1038 (100%), gaps = 2. *Poaceascomahalophilum* was found as a saprophyte on a decaying bamboo stick and its asexual morph is undetermined (Hyde et al. 2017). In terms of culture characteristics, *P.halophilum* differs from *P.zoysiiradicicola* in the slower vegetative growth rate on PDA (Hyde et al. 2017). Therefore, *P.zoysiiradicicola* sp. nov. was introduced in this study to accommodate CMML 20-50 and CMML 20-51 in the genus *Poaceascoma*.

### ﻿*In vitro* antifungal activity against *Rhizoctoniasolani* AG2-2(IIIB)

**Figure 27.**
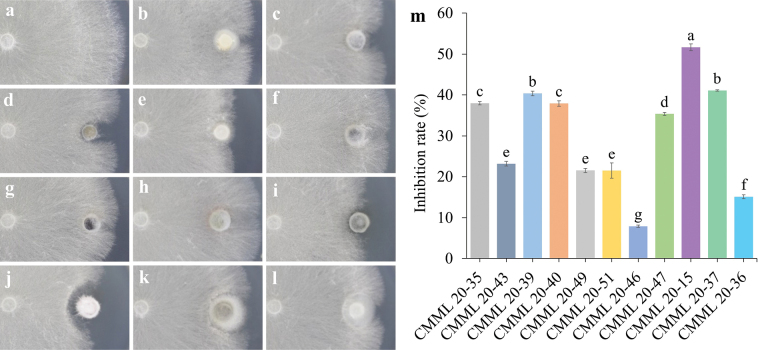
Dual culture of endophytic fungi against turfgrass brown patch pathogen *Rhizoctoniasolani* (AG-2-2 (IIIB)) **a** control **b***Dactylariahwasunensis* (CMML 20-35) **c***Lophiostomajeollanense* (CMML 20-43) **d***Magnaporthiopsiszoysiae* (CMML 20-39) **e***Niessliadimorphospora* (CMML 20-40) **f***Poaceascomaendophyticum* (CMML 20-49) **g***P.zoysiiradicicola* (CMML 20-51) **h***P.koreanum* (CMML 20-46) **i***P.magnum* (CMML 20-47) **j***Setophomazoysiae* (CMML 20-15) **k***Stagonosporaendophytica* (CMML 20-37) **l***Pseudorhypophilapoae* (CMML 20-36) **m** Inhibition rates of each fungal strains. Error bars indicate the standard errors of the means.

Antifungal activities of the species described above were tested by *in vitro* dual culture against *R.solani* AG2-2(IIIB), the casual pathogen of turf-grass brown patch disease. Different antifungal activities were observed amongst these species (Fig. 27a–l). Mycelial growth inhibition rates were 37.97% for *D.hwasunensis* CMML 20-35, 23.15% for *L.jeollanense* CMML 20-43, 40.35% for *M.zoysiae* CMML 20-39, 37.90% for *N.dimorphospora* CMML 20-40, 21.53% for *P.endophyticum* CMML 20-49, 21.51% for *P.zoysiiradicicola* CMML 20-51, 7.95% for *P.koreanum* CMML 20-46, 35.37% for *P.magnum* CMML 20-47, 51.68% for *S.zoysiae* CMML 20-15, 41.07% for *S.endophytica* CMML 20-37 and 15.12% for *P.poae* CMML 20-36 (Fig. 27m). *Setophomazoysiae* CMML 20-15 showed a significantly higher inhibition to *R.solani* AG2-2(IIIB) and was therefore selected for further antifungal activity assays.

### ﻿*In vitro* antifungal activity of *Setophomazoysiae* (CMML 20-15) against eight phytopathogens

**Figure 28.**
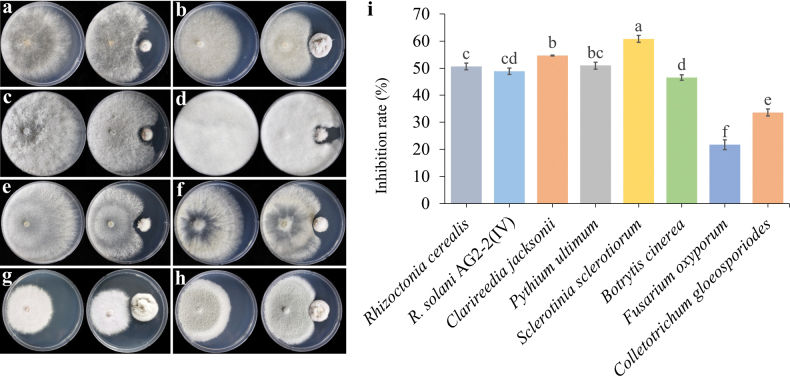
Dual culture of *Setophomazoysiae* (CMML20-15) against eight different plant pathogens **a***Rhizoctoniasolani* (AG-2-2(IV) KACC 40132) **b***R.cerealis* (KACC 40154) **c***Clarireediajacksonii* (CMML 20-31) **d***Pythiumultimum* (KACC 40705) **e***Sclerotiniasclerotiorum* (KACC 40457) **f***Botrytiscinerea* (CMML 20-BC04) **g***Fusariumoxysporum* (CMML 21-1) **h***Colletotrichumgloeosporiodes* (KACC 40003) **i** mycelial growth inhibition rates of each pathogen. Error bars indicate the standard errors of the means.

To test the antifungal spectrum of *S.zoysiae* (CMML 20-15), eight different agriculturally important phytopathogens were used for dual culture assay. Mycelial growth inhibition rates were 50.65% for *R.cerealis* (KACC 40154), 48.86% for *R.solani* (AG2-2(IV) KACC 40132), 54.70% for *Clarireediajacksonii* (CMML 20-31), 50.96% for *Pythiumultimum* (KACC 40705), 60.82% for *Sclerotiniasclerotiorum* (KACC 40457), 46.57% for *Botrytiscinerea* (CMML 20-BC04), 21.68% for *Fusariumoxysporum* (CMML 21-1) and 33.56% for *Colletotrichumgloeosporiodes* (KACC 40003) (Fig. 28).

### ﻿Antifungal activities of mycelial crude extracts of *Setophomazoysiae* (CMML 20-15)

**Figure 29.**
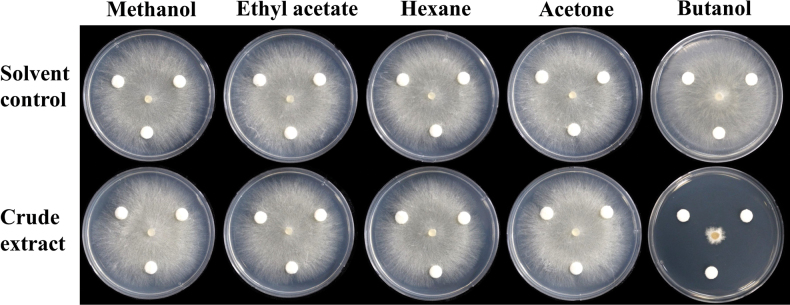
Antifungal activities of five crude extracts (methanol, ethyl acetate, hexane, acetone and butanol) of *Setophomazoysiae* (CMML20-15) against *Rhizoctoniasolani* (AG2-2(IIIB)) on PDA media. Paper discs were moistened with 20 μl of the crude extracts. Only solvent was treated in the control groups.

Mycelial crude extraction of *S.zoysiae* (CMML 20-15) was conducted using five different solvents (methanol, ethyl acetate, hexane, acetone and butanol). Antifungal activities of the five crude extracts against *R.solani* (AG2-2(IIIB)) were tested using a paper disc assay. No mycelial growth inhibition was found in methanol, ethyl acetate, hexane and acetone crude extracts, while dramatic growth inhibition was found in butanol extract (Fig. 29).

**Figure 30.**
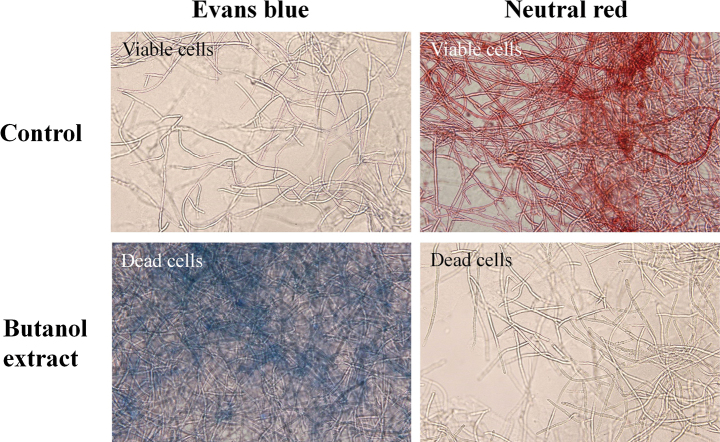
Mycelia of *Rhizoctoniasolani* (AG2-2(IIIB)) with or without exposure to butanol extract of *Setophomazoysiae* (CMML20-15) were stained with Evans blue and neutral red. Dead cells stained blue; viable cells, red.

To evaluate the mycelial viability of *R.solani* (AG2-2(IIIB)), Evans blue and neutral red were used to stain the mycelia from PDA plates with or without butanol extract of *S.zoysiae* (CMML 20-15). Staining results were examined under a microscope. Mycelia from the control groups (without exposure to butanol extract) were not stained by Evans blue, but were stained red by neutral red. On the contrary, mycelia exposed to butanol extract were stained blue by Evans blue, while remaining unstained by neutral red (Fig. 30). This revealed that cell death of *R.solani* (AG2-2(IIIB)) occurred under the treatment of butanol extract of *S.zoysiae* (CMML 20-15).

### ﻿*In planta* brown patch disease control

**Figure 31.**
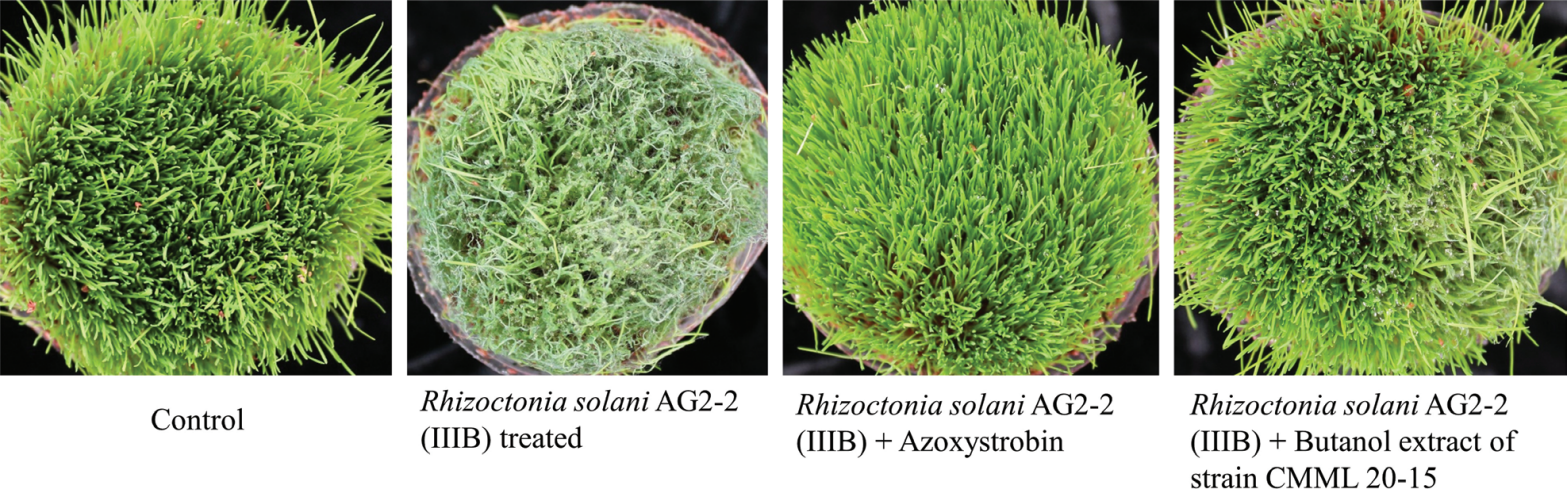
*In planta* brown patch control of creeping bentgrass by butanol extract of *Setophomazoysiae* (CMML20-15), with a comparison to a commercial fungicide azoxystrobin. Control group was treated with sterile water.

Butanol extract of *S.zoysiae* (CMML 20-15) was re-dissolved in sterile distilled water and used for brown patch control on creeping bentgrass in pots, with fungicide azoxystrobin used for the positive control. Symptoms of severe disease were observed on creeping bentgrass treated with only the pathogen *R.solani* (AG2-2(IIIB)). Fungicide azoxystrobin showed complete control of brown patch disease and only minor disease symptoms occurred on butanol extract-treated creeping bentgrass. No symptoms were observed in the sterile water-treated control group (Fig. 31).

## ﻿Discussion

In this study, both culture-independent and -dependent methods were used to determine endophytic fungal diversity associated with roots of *Z.japonica*. Novel species were identified based on morphological characterisation and multi-loci phylogenetic analyses. In addition, antifungal activities of the described species were tested against *R.solani* (AG2-2(IIIB)) with *S.zoysiae* (CMML20-15) being the best antagonist. Butanol crude extract of this strain was applied to control *R.solani* (AG2-2(IIIB)) by *in vitro* mycelial growth inhibition and *in planta* brown patch control. This is the first comprehensive work revealing fungal community on *Z.japonica* with an attempt for exploration and application of biological resources.

Through analysis of ITS amplicon reads, abundant OTUs were identified in roots of *Z.japonica*, dominated by members of the class *Sordariomycetes*. Fungi in this class have also been reported to dominate epiphytic and endophytic samples of tomato (Dong et al. 2021). The functions of endophytic fungi in this class and their roles in interacting with host plants deserve to be investigated. However, the classification of the most abundant taxa at the family and genus levels have remained unknown, suggesting that a large number of endophytic fungi in *Z.japonica* are remain unidentified. This supports the fact that endophytic fungi constitute a major part of unexplored fungal diversity (Rajamanikyam et al. 2017).

A variety of endophytic fungi were obtained in roots of *Z.japonica* by isolation in this study. Taxa in genera *Collectotrichum*, *Fusarium*, *Curvularia*, *Lophiostoma*, *Magnaporthiopsis*, *Poaceascoma* and *Stagonospora* were detected by both culture-independent and culture-dependent methods. Amongst these taxa, *Biappendiculispora* (currently *Lophiostoma*) and *Poaceascoma* were in the top 20 most abundant genera in ITS amplicon sequencing analysis and *Poaceascoma* spp. were also detected with high portions (18.52%) during isolation. Therefore, *Poaceascoma* spp. may have potential functions in the root-inhabiting mycobiome of *Z.japonica*.

Combining morphological characterisation and phylogenetic analysis represents a reliable strategy for fungal taxonomy. Based on this approach, a newly-recorded species (*Niessliadimorphospora*) in Korea and 10 new species (*Dactylariahwasunensis*, *Lophiostomajeollanense*, *Magnaporthiopsiszoysiae*, *Poaceascomaendophyticum*, *P.koreanum*, *P.magnum*, *P.zoysiiradicicola*, *Setophomazoysiae*, *Stagonosporaendophytica* and *Pseudorhypophilapoae*) were identified. Some genera such as *Magnaporthiopsis*, *Poaceascoma* and *Stagonospora* have been reported to be associated with grasses. Specifically, *Magnaporthiopsis* spp., such as *M.dharug*, *M.gadigal*, *M.gumbaynggirr* and *M.yugambeh*, were isolated from diseased turf-grass species in Australia (Wong et al. 2022). *Magnaporthiopsismeyeri-festucae* was associated with a summer patch-like disease of fescue turf-grasses in New Jersey (Luo et al. 2017). *Magnaporthiopsiszoysiae* was isolated from healthy roots of turf-grass *Z.japonica* in this study, it being genetically different from other *Magnaporthiopsis* spp. The genus *Poaceascoma* was established with type species *P.helicoides*, which is isolated as a saprophyte from dead stems of a grass in *Poaceae* (Phookamsak et al. 2015). Herein, four novel species *P.endophyticum*, *P.koreanum*, *P.magnum* and *P.zoysiiradicicola* were isolated as endophytes from turf-grass *Z.japonica*. Species in *Stagonospora* are commonly associated with grasses (Thambugala 2017) as pathogens and *S.endophytica* was supplemented as an endophyte in this study.

In previous studies, most of the endophytic fungi have not sporulated during culture. For example, Lin et al. (2007) reported that 48.9% of the obtained endophytic taxa from *Camptothecaacuminata* were non-sporulating fungi. Tejesvi et al. (2011) isolated 87 endophytic fungi from asymptomatic leaf tissues of *Rhododendrontomentosum* and most of the isolates were unable to sporulate. Therefore, it is difficult to identify endophytic non-sporulating fungi, based on morphological characters and sequence-based molecular tools can be used to achieve their classification (Promputtha et al. 2005; Huang et al. 2009; Tejesvi et al. 2011; Selim 2012). In the present study, species *L.jeollanense*, *P.endophyticum*, *P.koreanum*, *P.magnum* and *P.zoysiiradicicola* did not sporulate on synthetic media – only chlamydospores were produced. Similarly, sporulation was not observed in endophytic fungi, such as *Cyanodermellaasteris*, *Nemaniaaquilariae*, *N.yunnanensis*, *Batnamycesglobulariicola*, *Endopandanicolathailandica*, *Endomelanconiopsisfreycinetiae*, *Diaporthepandanicola* and *Mycoleptodiscusendophyticus*, which were proposed as novel species recently (Jahn et al. 2017; Tibpromma et al. 2018, 2021; Noumeur et al. 2020).

Endophytic fungi are also an important source of novel and potential bioactive compounds (Strobel and Daisy 2003; Tomita 2003; Rajamanikyam et al. 2017). Application of endophytic fungi as biocontrol agents has increased considerably. To date, endophytic fungi have been reported to control a variety of plant pathogens, ranging from pathogenic fungi, bacteria, oomycetes to plant-parasitic nematodes (Latz et al. 2018; Huang et al. 2020; Poveda et al. 2020; Hapida et al. 2021; Simamora et al. 2021). Brown patch caused by *R.solani* is a major disease of turf-grasses worldwide and its control relies heavily on fungicide applications (Daniels and Latin 2013). To reduce the use of fungicides, biocontrol agents have been applied for brown patch control, such as *Bacillusvelezensis* (GH1-13) (Lee et al. 2023), *Paenibacillusehimensis* (KWN38), *Stenotrophomonasmaltophilia* (C3) (Yuen and Zhang 2001) and *Trichodermaharzianum* (Guerrero et al. 2008). In this study, antifungal activities against *R.solani* (AG2-2(IIIB)) were observed amongst the tested endophytic fungi and *S.zoysiae* (CMML20-15) could serve as an antagonist for brown patch management. Additional dual culture assay suggested that *S.zoysiae* (CMML20-15) was potentially able to control the turf-grass large patch pathogen *R.solani* (AG2-2 (IV)), yellow patch pathogen *R.cerealis*, dollar spot pathogen *C.jacksonii*, Pythium blight pathogen *P.ultimum* and non-turf-grass pathogens *S.sclerotiorum*, *B.cinerea*, *F.oxysporum* and *C.gloeosporiodes*.

In conclusion, this study provides insights from the mycobiome diversity in *Z.japonica* to an application of a biocontrol agent in controlling pathogens of turf-grass, which will be valuable for future management of *Z.japonica*. Identifying the functions of other root-colonizing fungi of *Z.japonica* and searching for their bioactive compounds warrant future investigation.

## Supplementary Material

XML Treatment for Niesslia dimorphospora

XML Treatment for Dactylaria hwasunensis

XML Treatment for Magnaporthiopsis zoysiae

XML Treatment for Setophoma zoysiae

XML Treatment for Stagonospora endophytica

XML Treatment for Pseudorhypophila poae

XML Treatment for Lophiostoma jeollanense

XML Treatment for Poaceascoma magnum

XML Treatment for Poaceascoma endophyticum

XML Treatment for Poaceascoma koreanum

XML Treatment for Poaceascoma zoysiiradicicola

## ﻿Additional information

### Adherence to national and international regulations

All the fungal strains used in this study have been legally obtained, respecting the Convention on Biological Diversity (Rio Convention).

### Author ORCIDs

Haifeng Liu https://orcid.org/0000-0002-9733-9240

Hyeongju Choi https://orcid.org/0000-0003-0435-8483

Narayan Chandra Paul https://orcid.org/0000-0001-6568-515X

Hiran A. Ariyawansa https://orcid.org/0000-0001-8526-7721

Hyunkyu Sang https://orcid.org/0000-0002-7459-5217

### Data availability

The raw reads of ITS amplicon sequencing generated in this study were deposited in NCBI (BioProject no. PRJNA1165193). Nucleotide sequences of species described in this study were submitted to GenBank with accession numbers shown in Suppl. material 1.

## Supplementary materials

Supplementary material 1 Genbank accession numbers for the strains used for phylogenetic analysis in this study Data type xlsx https://binary.pensoft.net/file/1255389This dataset is made available under the Open Database License (http://opendatacommons.org/licenses/odbl/1.0/). The Open Database License (ODbL) is a license agreement intended to allow users to freely share, modify, and use this Dataset while maintaining this same freedom for others, provided that the original source and author(s) are credited.Haifeng Liu, Hyeongju Choi, Narayan Chandra Paul, Hiran A. Ariyawansa, Hyunkyu Sang

Supplementary material 2 Sequence alignments Data type zip Explanation note Sequence alignments (fas.-files in ZIP.archive). https://binary.pensoft.net/file/1255390This dataset is made available under the Open Database License (http://opendatacommons.org/licenses/odbl/1.0/). The Open Database License (ODbL) is a license agreement intended to allow users to freely share, modify, and use this Dataset while maintaining this same freedom for others, provided that the original source and author(s) are credited.Haifeng Liu, Hyeongju Choi, Narayan Chandra Paul, Hiran A. Ariyawansa, Hyunkyu Sang

## References

[B1] Abeywickrama P Qian N Jayawardena R Li Y Zhang W Guo K Zhang L Zhang G Yan J Li X Guo Z Hyde K Peng Y Zhao W (2023) Endophytic fungi in green manure crops; friends or foe? Mycosphere 14: 1–106. 10.5943/mycosphere/14/1/1

[B2] Andreasen M Skrede I Jaklitsch WM Voglmayr H Nordén B (2021) Multi-locus phylogenetic analysis of lophiostomatoid fungi motivates a broad concept of *Lophiostoma* and reveals nine new species. Persoonia 46: 240–271. 10.3767/persoonia.2021.46.09 35935892 PMC9311397

[B3] Andrews S (2018) FastQC: A quality control tool for high throughput sequence data. https://www.bioinformatics.babraham.ac.uk/projects/fastqc/

[B4] Bharadwaj R Jagadeesan H Kumar SR Ramalingam S (2020) Molecular mechanisms in grass-*Epichloë* interactions: Towards endophyte driven farming to improve plant fitness and immunity. World Journal of Microbiology and Biotechnology 36: 92. 10.1007/s11274-020-02868-5 32562008

[B5] Cai L Jeewon R Hyde KD (2006) Molecular systematics of *Zopfiella* and allied genera: Evidence from multi-gene sequence analyses. Mycological Research 110: 359–368. 10.1016/j.mycres.2006.01.007 16546361

[B6] Chandra A Milla‐Lewis S Yu Q (2017) An overview of molecular advances in zoysiagrass. Crop Science 57: S-73–S-81. 10.2135/cropsci2016.09.0822

[B7] Chen Z Jin Y Yao X Chen T Wei X Li C White JF Nan Z (2020) Fungal endophyte improves survival of *Loliumperenne* in low fertility soils by increasing root growth, metabolic activity and absorption of nutrients. Plant and Soil 452: 185–206. 10.1007/s11104-020-04556-7

[B8] Clay K (1990) Fungal endophytes of grasses. Annual review of Ecology and Systematics 21: 275–297. 10.1146/annurev.es.21.110190.001423

[B9] Crous PW Wingfield MJ Roux JJL Richardson DM Strasberg D Shivas RG Alvarado P Edwards J Moreno G Sharma R Sonawane MS Tan YP Altés A Barasubiye T Barnes CW Blanchette RA Boertmann D Bogo A Carlavilla JR Cheewangkoon R Daniel R De Beer ZW Yáñez-Morales MDJ Duong TA Fernández-Vicente J Geering ADW Guest DI Held BW Heykoop M Hubka V Ismail AM Kajale SC Khemmuk W Kolařík M Kurli R Lebeuf R Lévesque CA Lombard L Magista D Manjón JL Marincowitz S Mohedano JM Nováková A Oberlies NH Otto EC Paguigan ND Pascoe IG Pérez-Butrón JL Perrone G Rahi P Raja HA Rintoul T Sanhueza RMV Scarlett K Shouche YS Shuttleworth LA Taylor PWJ Thorn RG Vawdrey LL Solano-Vidal R Voitk A Wong PTW Wood AR Zamora JC Groenewald JZ (2015) Fungal Planet description sheets: 371–399. Persoonia 35: 264–327. 10.3767/003158515X690269 26823636 PMC4713108

[B10] Crous PW Wingfield MJ Burgess TI Hardy GEStJ Crane C Barrett S Cano-Lira JF Leroux JJ Thangavel R Guarro J Stchigel AM Martín MP Alfredo DS Barber PA Barreto RW Baseia IG Cano-Canals J Cheewangkoon R Ferreira RJ Gené J Lechat C Moreno G Roets F Shivas RG Sousa JO Tan YP Wiederhold NP Abell SE Accioly T Albizu JL Alves JL Antoniolli ZI Aplin N Araújo J Arzanlou M Bezerra JDP Bouchara J-P Carlavilla JR Castillo A Castroagudín VL Ceresini PC Claridge GF Coelho G Coimbra VRM Costa LA Da Cunha KC Da Silva SS Daniel R De Beer ZW Dueñas M Edwards J Enwistle P Fiuza PO Fournier J García D Gibertoni TB Giraud S Guevara-Suarez M Gusmão LFP Haituk S Heykoop M Hirooka Y Hofmann TA Houbraken J Hughes DP Kautmanová I Koppel O Koukol O Larsson E Latha KPD Lee DH Lisboa DO Lisboa WS López-Villalba Á Maciel JLN Manimohan P Manjón JL Marincowitz S Marney TS Meijer M Miller AN Olariaga I Paiva LM Piepenbring M Poveda-Molero JC Raj KNA Raja HA Rougeron A Salcedo I Samadi R Santos TAB Scarlett K Seifert KA Shuttleworth LA Silva GA Silva M Siqueira JPZ Souza-Motta CM Stephenson SL (2016) Fungal Planet description sheets: 469–557. Persoonia 37: 218–403. 10.3767/003158516X694499 28232766 PMC5315290

[B11] Crous PW Begoude BAD Boers J Braun U Declercq B Dijksterhuis J Elliott TF Garay-Rodriguez GA Jurjević Ž Kruse J Linde CC Loyd A Mound L Osieck ER Rivera-Vargas LI Quimbita AM Rodas CA Roux J Schumacher RK Starink-Willemse M Thangavel R Trappe JM Van Iperen AL Van Steenwinkel C Wells A Wingfield MJ Yilmaz N Groenewald JZ (2022a) New and interesting fungi. Fungal Systematics and Evolution 10: 19–90. 10.3114/fuse.2022.10.02 36789279 PMC9903348

[B12] Crous PW Boers J Holdom D Osieck Steinrucken TV Tan YP Vitelli JS Shivas RG Barrett M Boxshall A-G Broadbridge J Larsson E Lebel T Pinruan U Sommai S Alvarado P Bonito G Decock CA De La Peña-Lastra S Delgado G Houbraken J Maciá-Vicente JG Raja HA Rigueiro-Rodríguez A Rodríguez A Wingfield MJ Adams SJ Akulov A AL-Hidmi T Antonín V Arauzo S Arenas F Armada F Aylward J Bellanger J-M Berraf-Tebbal A Bidaud A Boccardo F Cabero J Calledda F Corriol G Crane JL Dearnaley JDW Dima B Dovana F Eichmeier A Esteve-Raventós F Fine M Ganzert L García D Torres-Garcia D Gené J Gutiérrez A Iglesias P Istel Ł Jangsantear P Jansen GM Jeppson M Karun NC Karich A Khamsuntorn P Kokkonen K Kolarík M Kubátová A Labuda R Lagashetti AC Lifshitz N Linde C Loizides M Luangsa-ard JJ Lueangjaroenkit P Mahadevakumar S Mahamedi AE Malloch DW Marincowitz S Mateos A Moreau P-A Miller AN Molia A Morte A Navarro-Ródenas A Nebesářová J Nigrone E Nuthan BR Oberlies NH Pepori AL Rämä T Rapley D Reschke K Robicheau BM Roets F Roux J Saavedra M Sakolrak B Santini A Ševčíková H Singh PN Singh SK Somrithipol S Spetik M Sridhar KR Starink-Willemse M Taylor VA Van Iperen AL Vauras J Walker AK Wingfield BD Yarden O Cooke AW Manners AG Pegg KG Groenewald JZ (2022b) Fungal Planet description sheets: 1383–1435. Persoonia 48: 261–371. 10.3767/persoonia.2022.48.08 38234686 PMC10792288

[B13] Cubero OF Crespo A Fatehi J Bridge PD (1999) DNA extraction and PCR amplification method suitable for fresh, herbarium-stored, lichenized, and other fungi. Plant Systematics and Evolution 216: 243–249. 10.1007/BF01084401

[B14] Daniels JP Latin R (2013) Residual efficacy of fungicides for controlling brown patch on creeping bentgrass fairways. Plant Disease 97: 1620–1625. 10.1094/PDIS-12-12-1130-RE 30716863

[B15] De Hoog G (1985) Taxonomy of the *Dactylaria* complex, VI. Key to the genera and checklist of epithets. Studies in Mycology 26: 97–122.

[B16] Dong C Wang L Li Q Shang Q (2021) Epiphytic and endophytic fungal communities of tomato plants. Horticultural Plant Journal 7: 38–48. 10.1016/j.hpj.2020.09.002

[B17] Edgar RC (2016) SINTAX: a simple non-Bayesian taxonomy classifier for 16S and ITS sequences. Bioinformatics. Preprint at bioRxiv. 10.1101/074161

[B18] Fávaro LCDL Sebastianes FLDS Araújo WL (2012) *Epicoccumnigrum* P16, a sugarcane endophyte, produces antifungal compounds and induces root growth. PLOS ONE 7: e36826. 10.1371/journal.pone.0036826 PMC3366970 22675473

[B19] Ge Y Norton T Wang Z-Y (2006) Transgenic zoysiagrass (*Zoysiajaponica*) plants obtained by agrobacterium-mediated transformation. Plant Cell Reports 25: 792–798. 10.1007/s00299-006-0123-8 16523287

[B20] Glass NL Donaldson GC (1995) Development of primer sets designed for use with the PCR to amplify conserved genes from filamentous ascomycetes. Applied and Environmental Microbiology 61: 1323–1330. 10.1128/aem.61.4.1323-1330.1995 7747954 PMC167388

[B21] Guerrero C Vitoriano J Neto L Dionísio L (2008) Control of fungi diseases on turfgrass using *Trichodermaharzianum*. Wseas Transactions on Environment and Development 9: 736–754.

[B22] Hapida Y Elfita E Widjajanti H Salni S (2021) Biodiversity and antibacterial activity of endophytic fungi isolated from jambu bol (*Syzygiummalaccense*). Biodiversitas Journal of Biological Diversity 22: 5668–5677. 10.13057/biodiv/d221253

[B23] Harms K Milic A Stchigel AM Stadler M Surup F Marin-Felix Y (2021) Three new derivatives of zopfinol from *Pseudorhypophilamangenotii* gen. et comb. nov. Journal of Fungi 7: 181. 10.3390/jof7030181 PMC8000789 33802411

[B24] Hassen H (1929) Etiology of the pink-root disease of onions. Phytopathology 19: 691–704.

[B25] Held DW Potter DA (2012) Prospects for managing turfgrass pests with reduced chemical inputs. Annual Review of Entomology 57: 329–354. 10.1146/annurev-ento-120710-100542 21910640

[B26] Huang L-Q Niu Y-C Su L Deng H Lyu H (2020) The potential of endophytic fungi isolated from cucurbit plants for biocontrol of soilborne fungal diseases of cucumber. Microbiological Research 231: 126369. 10.1016/j.micres.2019.126369 31733598

[B27] Huang W Cai Y Surveswaran S Hyde K Corke H Sun M (2009) Molecular phylogenetic identification of endophytic fungi isolated from three *Artemisia* species. Fungal Diversity: 69–88.

[B28] Hyde KD Norphanphoun C Abreu VP Bazzicalupo A Thilini Chethana KW Clericuzio M Dayarathne MC Dissanayake AJ Ekanayaka AH He M-Q Hongsanan S Huang S-K Jayasiri SC Jayawardena RS Karunarathna A Konta S Kušan I Lee H Li J Lin C-G Liu N-G Lu Y-Z Luo Z-L Manawasinghe IS Mapook A Perera RH Phookamsak R Phukhamsakda C Siedlecki I Soares AM Tennakoon DS Tian Q Tibpromma S Wanasinghe DN Xiao Y-P Yang J Zeng X-Y Abdel-Aziz FA Li W-J Senanayake IC Shang Q-J Daranagama DA De Silva NI Thambugala KM Abdel-Wahab MA Bahkali AH Berbee ML Boonmee S Bhat DJ Bulgakov TS Buyck B Camporesi E Castañeda-Ruiz RF Chomnunti P Doilom M Dovana F Gibertoni TB Jadan M Jeewon R Jones EBG Kang J-C Karunarathna SC Lim YW Liu J-K Liu Z-Y Plautz HL Lumyong S Maharachchikumbura SSN Matočec N McKenzie EHC Mešić A Miller D Pawłowska J Pereira OL Promputtha I Romero AI Ryvarden L Su H-Y Suetrong S Tkalčec Z Vizzini A Wen T-C Wisitrassameewong K Wrzosek M Xu J-C Zhao Q Zhao R-L Mortimer PE (2017) Fungal diversity notes 603–708: Taxonomic and phylogenetic notes on genera and species. Fungal Diversity 87: 1–235. 10.1007/s13225-017-0391-3

[B29] Jahn L Schafhauser T Pan S Weber T Wohlleben W Fewer D Sivonen K Flor L Van Pée K-H Caradec T Jacques P Huijbers Mieke ME Van Berkel Willem JH Ludwig-Müller J (2017) *Cyanodermellaasteris* sp. nov. (*Ostropales*) from the inflorescence axis of *Astertataricus*. Mycotaxon 132: 107–123. 10.5248/132.107

[B30] Jha P Kaur T Chhabra I Panja A Paul S Kumar V Malik T (2023) Endophytic fungi: hidden treasure chest of antimicrobial metabolites interrelationship of endophytes and metabolites. Frontiers in Microbiology 14: 1227830. 10.3389/fmicb.2023.1227830 PMC10366620 37497538

[B31] Kaiser W Ndimande B Hawksworth D (1979) Leaf-scorch disease of sugarcane in Kenya caused by a new species of *Leptosphaeria*. Mycologia 71: 479–492. 10.1080/00275514.1979.12021031

[B32] Kalyaanamoorthy S Minh BQ Wong TKF Von Haeseler A Jermiin LS (2017) ModelFinder: fast model selection for accurate phylogenetic estimates. Nature Methods 14: 587–589. 10.1038/nmeth.4285 28481363 PMC5453245

[B33] Khidir HH Eudy DM Porras-Alfaro A Herrera J Natvig DO Sinsabaugh RL (2010) A general suite of fungal endophytes dominate the roots of two dominant grasses in a semiarid grassland. Journal of Arid Environments 74: 35–42. 10.1016/j.jaridenv.2009.07.014

[B34] Latz MAC Jensen B Collinge DB Jørgensen HJL (2018) Endophytic fungi as biocontrol agents: elucidating mechanisms in disease suppression. Plant Ecology & Diversity 11: 555–567. 10.1080/17550874.2018.1534146

[B35] Lee G Choi H Liu H Han Y-H Paul NC Han GH Kim H Kim PI Seo S-I Song J Sang H (2023) Biocontrol of the causal brown patch pathogen *Rhizoctoniasolani* by *Bacillusvelezensis* GH1-13 and development of a bacterial strain specific detection method. Frontiers in Plant Science 13: 1091030. 10.3389/fpls.2022.1091030 PMC9868939 36699832

[B36] Lin X Lu C Huang Y Zheng Z Su W Shen Y (2007) Endophytic fungi from a pharmaceutical plant, *Camptothecaacuminata*: isolation, identification and bioactivity. World Journal of Microbiology and Biotechnology 23: 1037–1040. 10.1007/s11274-006-9329-8

[B37] Liu F Wang J Li H Wang W Cai L (2019) *Setophoma* spp. on *Camelliasinensis*. Fungal Systematics and Evolution 4: 43–57. 10.3114/fuse.2019.04.05 32467906 PMC7241682

[B38] Liu YJ Whelen S Hall BD (1999) Phylogenetic relationships among ascomycetes: evidence from an RNA polymerse II subunit. Molecular Biology and Evolution 16: 1799–1808. 10.1093/oxfordjournals.molbev.a026092 10605121

[B39] Loch DS Ebina M Choi JS Han L (2017) Ecological implications of *Zoysia* species, distribution, and adaptation for management and use of zoysiagrasses. International Turfgrass Society Research Journal 13: 11–25. 10.2134/itsrj2016.10.0857

[B40] Luo J Vines PL Grimshaw A Hoffman L Walsh E Bonos SA Clarke BB Murphy JA Meyer WA Zhang N (2017) *Magnaporthiopsismeyeri-festucae* sp. nov., associated with a summer patch-like disease of fine fescue turfgrasses. Mycologia 109: 780–789. 10.1080/00275514.2017.1400306 29293408

[B41] Luo Z-L Bahkali AH Liu X-Y Phookamsak R Zhao Y-C Zhou D-Q Su H-Y Hyde KD (2016) *Poaceascomaaquaticum* sp. nov. (*Lentitheciaceae*), a new species from submerged bamboo in freshwater. Phytotaxa 253: 71. 10.11646/phytotaxa.253.1.5

[B42] Manzotti A Bergna A Burow M Jørgensen HJL Cernava T Berg G Collinge DB Jensen B (2020) Insights into the community structure and lifestyle of the fungal root endophytes of tomato by combining amplicon sequencing and isolation approaches with phytohormone profiling. FEMS Microbiology Ecology 96: fiaa052. 10.1093/femsec/fiaa052 PMC7174037 32239208

[B43] Marin-Felix Y Miller AN Cano-Lira JF Guarro J García D Stadler M Huhndorf SM Stchigel AM (2020) Re-evaluation of the order *Sordariales*: Delimitation of *Lasiosphaeriaceae* s. str., and introduction of the new families *Diplogelasinosporaceae*, *Naviculisporaceae*, and *Schizotheciaceae*. Microorganisms 8: 1430. 10.3390/microorganisms8091430 PMC7565071 32957559

[B44] Martin M (2011) Cutadapt removes adapter sequences from high-throughput sequencing reads. EMBnet. journal 17: 10–12. 10.14806/ej.17.1.200

[B45] Matheny PB Liu YJ Ammirati JF Hall BD (2002) Using RPB1 sequences to improve phylogenetic inference among mushrooms (*Inocybe*, *Agaricales*). American Journal of Botany 89: 688–698. 10.3732/ajb.89.4.688 21665669

[B46] McMurdie PJ Holmes S (2013) phyloseq: An R package for reproducible interactive analysis and graphics of microbiome census data. PLOS ONE 8: e61217. 10.1371/journal.pone.0061217 PMC3632530 23630581

[B47] Meyer WA Torres MS White JF (2015) Biology and applications of fungal endophytes in turfgrasses. In: Stier JC Horgan BP Bonos SA (Eds) Turfgrass: biology, use, and management.American Society of Agronomy, Madison, WI, USA, 713–731. 10.2134/agronmonogr56.c20

[B48] Monard C Gantner S Stenlid J (2013) Utilizing ITS1 and ITS2 to study environmental fungal diversity using pyrosequencing. FEMS Microbiology Ecology 84: 165–175. 10.1111/1574-6941.12046 23176677

[B49] Nguyen L-T Schmidt HA Von Haeseler A Minh BQ (2015) IQ-TREE: A fast and effective stochastic algorithm for estimating maximum-likelihood phylogenies. Molecular Biology and Evolution 32: 268–274. 10.1093/molbev/msu300 25371430 PMC4271533

[B50] Nilsson RH Larsson K-H Taylor AFS Bengtsson-Palme J Jeppesen TS Schigel D Kennedy P Picard K Glöckner FO Tedersoo L Saar I Kõljalg U Abarenkov K (2019) The UNITE database for molecular identification of fungi: Handling dark taxa and parallel taxonomic classifications. Nucleic Acids Research 47: D259–D264. 10.1093/nar/gky1022 PMC6324048 30371820

[B51] Noumeur SR Teponno RB Helaly SE Wang X-W Harzallah D Houbraken J Crous PW Stadler M (2020) Diketopiperazines from *Batnamycesglobulariicola*, gen. & sp. nov. (*Chaetomiaceae*), a fungus associated with roots of the medicinal plant *Globulariaalypum* in Algeria. Mycological Progress 19: 589–603. 10.1007/s11557-020-01581-9

[B52] O’Donnell K Cigelnik E (1997) Two divergent intragenomic rDNA ITS2 types within a monophyletic lineage of the fungus *Fusarium* are nonorthologous. Molecular Phylogenetics and Evolution 7: 103–116. 10.1006/mpev.1996.0376 9007025

[B53] Oksanen J Simpson GL Blanchet FG Kindt R Legendre P Minchin PR O’Hara RB Solymos P Stevens MHH Szoecs E Wagner H Barbour M Bedward M Bolker B Borcard D Carvalho G Chirico M De Caceres M Durand S Evangelista HBA FitzJohn R Friendly M Furneaux B Hannigan G Hill MO Lahti L McGlinn D Ouellette M-H Cunha ER Smith T Stier A Ter Braak CJF Weedon J (2022) vegan: Community ecology package. https://cran.r-project.org/web/packages/vegan/index.html

[B54] Phookamsak R Manamgoda DS Li W-J Dai D-Q Singtripop C Hyde KD (2015) *Poaceascomahelicoides* gen et sp. nov., a new genus with *scolecospores* in *Lentitheciaceae*. Cryptogamie, Mycologie 36: 225–236. 10.7872/crym/v36.iss2.2015.225

[B55] Porras-Alfaro A Herrera J Sinsabaugh RL Odenbach KJ Lowrey T Natvig DO (2008) Novel root fungal consortium associated with a dominant desert grass. Applied and Environmental Microbiology 74: 2805–2813. 10.1128/AEM.02769-07 18344349 PMC2394874

[B56] Poveda J Abril-Urias P Escobar C (2020) Biological control of plant-parasitic nematodes by filamentous fungi inducers of resistance: *Trichoderma*, mycorrhizal and endophytic fungi. Frontiers in Microbiology 11: 992. 10.3389/fmicb.2020.00992 PMC7261880 32523567

[B57] Poveda J Eugui D Abril-Urías P Velasco P (2021) Endophytic fungi as direct plant growth promoters for sustainable agricultural production. Symbiosis 85: 1–19. 10.1007/s13199-021-00789-x

[B58] Promputtha I Jeewon R Lumyong S McKenzie EH Hyde KD (2005) Ribosomal DNA fingerprinting in the identification of non sporulating endophytes from *Magnolialiliifera* (*Magnoliaceae*). Fungal Diversity 20: 167–186.

[B59] Quaedvlieg W Verkley GJM Shin H-D Barreto RW Alfenas AC Swart WJ Groenewald JZ Crous PW (2013) Sizing up *Septoria*. Studies in Mycology 75: 307–390. 10.3114/sim0017 24014902 PMC3713890

[B60] Rajamanikyam M Vadlapudi V Amanchy R Upadhyayula SM (2017) Endophytic fungi as novel resources of natural therapeutics. Brazilian Archives of Biology and Technology 60. 10.1590/1678-4324-2017160542

[B61] Rajani P Rajasekaran C Vasanthakumari MM Olsson SB Ravikanth G Uma Shaanker R (2021) Inhibition of plant pathogenic fungi by endophytic *Trichoderma* spp. through mycoparasitism and volatile organic compounds. Microbiological Research 242: 126595. 10.1016/j.micres.2020.126595 33017769

[B62] Rehner SA Buckley E (2005) A *Beauveria* phylogeny inferred from nuclear ITS and EF1-α sequences: evidence for cryptic diversification and links to *Cordyceps* teleomorphs. Mycologia 97: 84–98. 10.1080/15572536.2006.11832842 16389960

[B63] Ripa FA Cao W Tong S Sun J (2019) Assessment of plant growth promoting and abiotic stress tolerance properties of wheat endophytic fungi. BioMed Research International 2019: 1–12. 10.1155/2019/6105865 PMC6457323 31032353

[B64] Rognes T Flouri T Nichols B Quince C Mahé F (2016) VSEARCH: a versatile open source tool for metagenomics. PeerJ 4: e2584. 10.7717/peerj.2584 PMC5075697 27781170

[B65] Ronquist F Teslenko M Van Der Mark P Ayres DL Darling A Höhna S Larget B Liu L Suchard MA Huelsenbeck JP (2012) MrBayes 3.2: Efficient Bayesian phylogenetic inference and model choice across a large model space. Systematic Biology 61: 539–542. 10.1093/sysbio/sys029 22357727 PMC3329765

[B66] Schmitt I Crespo A Divakar PK Fankhauser JD Herman-Sackett E Kalb K Nelsen MP Nelson NA Rivas-Plata E Shimp AD Widhelm T Lumbsch HT (2009) New primers for promising single-copy genes in fungal phylogenetics and systematics. Persoonia 23: 35–40. 10.3767/003158509X470602 20198159 PMC2802727

[B67] Selim K (2012) Biology of endophytic fungi. Current Research in Environmental & Applied Mycology 2: 31–82. 10.5943/cream/2/1/3

[B68] Simamora AV Hahuly MV Henuk JB (2021) Endophytic fungi as potential biocontrol agents of *Phytophthorapalmivora* in the cocoa plant. Biodiversitas Journal of Biological Diversity 22: 2601–2609. 10.13057/biodiv/d220519

[B69] Soreng RJ Peterson PM Romaschenko K Davidse G Zuloaga FO Judziewicz EJ Filgueiras TS Davis JI Morrone O (2015) A worldwide phylogenetic classification of the Poaceae (Gramineae). Journal of Systematics and Evolution 53: 117–137. 10.1111/jse.12150

[B70] Sridhar KR (2019) Diversity, ecology, and significance of fungal endophytes. In: Jha S (Ed.) Endophytes and secondary metabolites.Reference Series in Phytochemistry. Springer, Cham, 61–100. 10.1007/978-3-319-90484-9_5

[B71] Stiller JW Hall BD (1997) The origin of red algae: Implications for plastid evolution. Proceedings of the National Academy of Sciences 94: 4520–4525. 10.1073/pnas.94.9.4520 PMC20755 9114022

[B72] Strobel G Daisy B (2003) Bioprospecting for microbial endophytes and their natural products. Microbiology and Molecular Biology Reviews 67: 491–502. 10.1128/MMBR.67.4.491-502.2003 14665674 PMC309047

[B73] Tan Y Marney T Bishop-Hurley S Bransgrove K Shivas R (2021) Index Fungorum no. 490.

[B74] Tanney JB Di Stefano J Miller JD McMullin DR (2023) Natural products from the *Picea* foliar endophytes *Niessliaendophytica* sp. nov. and *Strasseriageniculata*. Mycological Progress 22: 17. 10.1007/s11557-023-01869-6

[B75] Tejesvi MV Kajula M Mattila S Pirttilä AM (2011) Bioactivity and genetic diversity of endophytic fungi in *Rhododendrontomentosum* Harmaja. Fungal Diversity 47: 97–107. 10.1007/s13225-010-0087-4

[B76] Thambugala K (2017) Mycosphere notes 1–50: Grass (*Poaceae*) inhabiting *Dothideomycetes*. Mycosphere 8: 697–796. 10.5943/mycosphere/8/4/13

[B77] Thambugala KM Hyde KD Tanaka K Tian Q Wanasinghe DN Ariyawansa HA Jayasiri SC Boonmee S Camporesi E Hashimoto A Hirayama K Schumacher RK Promputtha I Liu Z-Y (2015) Towards a natural classification and backbone tree for *Lophiostomataceae*, *Floricolaceae*, and *Amorosiaceae* fam. nov. Fungal Diversity 74: 199–266. 10.1007/s13225-015-0348-3

[B78] Tibpromma S Hyde KD Bhat JD Mortimer PE Xu J Promputtha I Doilom M Yang J-B Tang AMC Karunarathna SC (2018) Identification of endophytic fungi from leaves of *Pandanaceae* based on their morphotypes and DNA sequence data from southern Thailand. MycoKeys 33: 25–67. 10.3897/mycokeys.33.23670 PMC6283267 30532625

[B79] Tibpromma S Zhang L Karunarathna SC Du T-Y Phukhamsakda C Rachakunta M Suwannarach N Xu J Mortimer PE Wang Y-H (2021) Volatile constituents of endophytic fungi isolated from *Aquilariasinensis* with descriptions of two new species of *Nemania*. Life 11: 363. 10.3390/life11040363 PMC8073270 33921887

[B80] Tiwari P Kang S Bae H (2023) Plant-endophyte associations: Rich yet under-explored sources of novel bioactive molecules and applications. Microbiological Research 266: 127241. 10.1016/j.micres.2022.127241 36272324

[B81] Tomita F (2003) Endophytes in southeast Asia and Japan: Their taxonomic diversity and potential applications. Fungal Diversity 14: 187–204.

[B82] Tothill JC Hacker JB (1983) The grasses of southern Queensland. University of Queensland Press, St. Lucia, Queensland, Australia.

[B83] Vilgalys R Hester M (1990) Rapid genetic identification and mapping of enzymatically amplified ribosomal DNA from several *Cryptococcus* species. Journal of Bacteriology 172: 4238–4246. 10.1128/jb.172.8.4238-4246.1990 2376561 PMC213247

[B84] Vines PL Hoffmann FG Meyer F Allen TW Luo J Zhang N Tomaso-Peterson M (2020) *Magnaporthiopsiscynodontis*, a novel turfgrass pathogen with widespread distribution in the United States. Mycologia 112: 52–63. 10.1080/00275514.2019.1676614 31846602

[B85] White TJ Bruns T Lee S Taylor J (1990) Amplification and direct sequencing of fungal ribosomal RNA genes for phylogenetics. In: Innis MA Gelfand DH Sninsky JJ White TJ (Eds) PCR protocols: a guide to methods and applications.Academic Press, San Diego, 315–322. 10.1016/B978-0-12-372180-8.50042-1

[B86] Wickham H (2011) ggplot2. WIREs Computational Statistics 3: 180–185. 10.1002/wics.147

[B87] Wiewióra B Żurek G Żurek M (2015) Endophyte-mediated disease resistance in wild populations of perennial ryegrass (*Loliumperenne*). Fungal Ecology 15: 1–8. 10.1016/j.funeco.2015.01.004

[B88] Wong P Tan Y Weese T Shivas R (2022) *Magnaporthiopsis* species associated with patch diseases of turfgrasses in Australia. Mycosphere 13: 602–611. 10.5943/mycosphere/13/1/5

[B89] Xia Y Sahib MR Amna A Opiyo SO Zhao Z Gao YG (2019) Culturable endophytic fungal communities associated with plants in organic and conventional farming systems and their effects on plant growth. Scientific Reports 9: 1669. 10.1038/s41598-018-38230-x PMC6368545 30737459

[B90] Yang D-H Sun H-J Jeong O-C Jin I-D Kang H-G Lee H-Y (2023) Development of ‘Halla Green 12’ cultivar of zoysiagrass, a hybrid of *Z.matrella* and *Z.japonica*. Korean Journal of Breeding Science 55: 147–155. 10.9787/KJBS.2023.55.2.147

[B91] Yuen G Zhang Z (2001) Control of brown patch disease using the bacterium *Stenotrophomonasmaltophilia* strain C3 and culture fluid. International Turfgrass Society Research Journal 9: 742–747.

[B92] Zeng X Tan T Tian F Wang Y Wen T (2023) OFPT: a one-stop software for fungal phylogeny. Mycosphere 14: 1730–1741. 10.5943/mycosphere/14/1/20

[B93] Zhang J-F Liu J-K Hyde KD Chen Y-Y Ran H-Y Liu Z-Y (2023) *Ascomycetes* from karst landscapes of Guizhou Province, China. Fungal Diversity 122: 1–160. 10.1007/s13225-023-00524-5

[B94] Zhang N Zhao S Shen Q (2011) A six-gene phylogeny reveals the evolution of mode of infection in the rice blast fungus and allied species. Mycologia 103: 1267–1276. 10.3852/11-022 21642347

